# Astrocytes contribute to olanzapine-mediated reversal of kleefstra syndrome-associated neurodevelopmental regression

**DOI:** 10.1172/JCI195803

**Published:** 2026-06-30

**Authors:** Karlijn Vermeulen-Kalk, Shan Wang, Joost Kummeling, Britt Mossink, Kim Wijnant, Carlos Gonzales Jiménez, Zoe Frazier, Brian Rozumny, Anne O’Donnell-Luria, Ellen Hanson, Monica Frega, Katrin Linda, Moritz Negwer, Bas Lendemeijer, Astrid Oudakker, Monica Pop-Purceleanu, Joost Janzing, Linde van Dongen, Femke M.S. de Vrij, Steven A. Kushner, Ilse van der Werf, Chantal Schoenmaker, Wouter Oomens, Siddharth Srivastava, Jos I.M. Egger, Hans van Bokhoven, Dirk Schubert, Nael Nadif Kasri, Tjitske Kleefstra

**Affiliations:** 1 Department of Human Genetics, Radboud UMC, Nijmegen, Netherlands.; 2 Centre of Excellence for Neuropsychiatry, Vincent van Gogh Institute for Psychiatry, Venray, Netherlands.; 3 Department for Intellectual Disabilities, Karakter Child and Adolescent Psychiatry, Ede, Netherlands.; 4 Donders Institute for Brain, Cognition and Behaviour, Nijmegen, Netherlands.; 5 Rosamund Stone Zander Translational Neuroscience Center, Department of Neurology,; 6 Division of Developmental Medicine,; 7 Division of Genetics and Genomics, Boston Children’s Hospital, Boston, Massachusetts, USA.; 8 Program in Medical and Population Genetics, Broad Institute of MIT and Harvard, Cambridge, Massachusetts, USA.; 9 Department of Neurology, Boston Children’s Hospital, Boston, Massachusetts, USA.; 10 Department of Psychiatry, Erasmus MC University Medical Center, Rotterdam, Netherlands.; 11 Department of Psychiatry, Radboud University Medical Center, Nijmegen, Netherlands.; 12 ENCORE Expertise Center for Neurodevelopmental Disorders, Erasmus MC, Rotterdam, Netherlands.; 13 Department of Psychiatry, Columbia University, New York, New York, USA.; 14 Department of Medical Neurosciences, Radboud UMC, Nijmegen, Netherlands.; 15 Stevig Specialized and Forensic Care for People with Intellectual Disabilities, Dichterbij, Oostrum, Netherlands.; 16 Department of Clinical Genetics, Erasmus MC University Medical Center, Rotterdam, Netherlands.

**Keywords:** Development, Neuroscience, Monogenic diseases, Neurodevelopment, iPS cells

## Abstract

Kleefstra syndrome (KLEFS1) results from *EHMT1* haploinsufficiency and is characterized by variable neurodevelopmental delays and psychopathology. Developmental regression, marked by the sudden loss of previously acquired daily life skills during late puberty or early adulthood, has emerged as a severe complication in individuals with KLEFS1. To investigate the clinical and molecular mechanisms underlying developmental regression and assess the therapeutic potential of olanzapine, we conducted a sequential study in an international cohort of 54 individuals with KLEFS1. Among 16 individuals treated with olanzapine, 10 exhibited a beneficial response based upon improvement of their adaptive functioning, and 4 showed temporary improvement. These clinical findings informed preclinical studies using human induced pluripotent stem cell-derived and ex-vivo cortical slices from a mouse model of KLEFS1. We identified hyperactivity in *EHMT1*^+/–^ neuronal networks cocultured with *EHMT1^+/–^* astrocytes, a dysfunction reversible by olanzapine. Mechanistically, *EHMT1^+/–^* astrocytes displayed elevated levels of S100B, a neuroinflammatory marker contributing to neuronal network hyperactivity. Notably, olanzapine treatment reduced S100B levels, and pharmacological inhibition or genetic knockdown of *S100B* in *EHMT1*^+/–^ astrocytes was sufficient to rescue the neuronal hyperactivity phenotype. These findings underscore a critical role for astrocytes in KLEFS1 pathophysiology and identify a potential cellular target for olanzapine in mitigating developmental regression.

## Abstract 2

Kleefstra syndrome (KLEFS1) involves neurodevelopmental regression. Olanzapine improved symptoms in some patients, potentialy by reducing astrocyte-driven brain hyperactivity linked to elevated S100β levels.

## Introduction

Kleefstra syndrome (KLEFS1) is a genetic neurodevelopmental disorder with a prevalence of approximately 1 in 35,000 individuals, caused by loss of function of Euchromatic histone methyltransferase 1 (*EHMT1*) (KLEFS1; OMIM #610253) (1). The protein product EHMT1, also known as G9a like Protein (GLP), together with EHMT2 (G9a), is crucial in controlling gene expression in numerous tissues and biological processes. The phenotype of KLEFS1 is broad, comprising developmental delay (DD)/intellectual disability (ID), autism spectrum disorders (ASD), abnormal growth and metabolism, and various other somatic and neuropsychiatric features (2–5).

In addition to the developmental delays, individuals with KLEFS1 seem highly vulnerable to develop psychiatric disorders, such as major depressive disorders, anxiety, and psychotic disorders (6, 7) (Supplemental Table 1; supplemental material available online with this article; https://doi.org/10.1172/JCI195803DS1). A major devastating aspect of the neuropsychiatric disease course comprises a sudden decline in functionality, which is increasingly observed in the KLEFS1 cohorts during adolescence and adulthood but difficult to characterize and define. We recently defined ‘regressive episodes’ as decline of functioning in at least one of the adaptive behavior domains of (a) practical and/or (b) conceptual and/or (c) social-emotional skills in the individual, and, when left untreated would last at least several months (8). Typically, these episodes manifest in KLEFS1 with sudden and severe sleep problems usually accompanied by symptoms of mood disorder and/or psychotic symptoms (9). In our previous studies and scarce case reports published, no clear somatic substrate has been identified. We hypothesize that the complex neuropsychiatric symptomatology is the result of a loss of brain homeostasis by yet unknown clear causes and has a detrimental impact on daily life functioning, on individual as well as family and societal level (10). Luxating factors have been described as stressors of somatic, psychological, and social origin (Supplemental Table 2, (9, 11–13)).

We aimed to address the urgent need in characterizing the course of this devastating symptomatology and establish an intervention strategy to treat the regressive episodes as soon as suspected upon clinical symptomatology. To achieve this, we continued our natural history study (14) in an extended international cohort of KLEFS1 individuals above 12 years of biological age. We applied a systematic approach by combining careful neuropsychiatric examination with an established test battery comprising various measures and questionnaires, enabling repeated data collection in a sequential design. Regarding the need for treatment of the severe episodes of loss-of-functioning, we designed a pharmacological intervention strategy on case-based experiences both by our clinical experience and that of others (Supplemental Table 2). Based on these data, we selected olanzapine as the preferred candidate drug of choice for intervention in our study population. We examined whether olanzapine is effective to treat regressive episodes in KLEFS1 by measuring adaptive functioning in everyone over time as the primary outcome, added by data collection on optimal dosage regime, tolerability, and duration of treatment (Supplemental Table 3).

To understand the pathogenesis of KLEFS1 and develop therapeutic strategies, it is crucial to define the phenotypes of *EHMT1* deficiency in the main brain cell types that express EHMT1. Its expression in the brain has been shown in neurons and glial cells, with the highest expression in astrocytes (15). Previous research mostly focused on the role of *EHMT1* in neurons and microglia. *EHMT1* deficiency in rat and mouse cortical cultures in vitro caused a reduced neuronal network synchronization (16), increased excitation/inhibition balance (17), and impaired homeostatic synaptic plasticity (18). *Ehmt1*^+/–^ mouse brain displayed an increased number of activated microglia, which affected dendritic spines (19). Notably, *Ehmt1*-deficient mouse cortical cultures containing astrocytes and neurons showed a hyperactive network phenotype (17), whereas no hyperactive phenotype could be observed in *EHMT1*^+/–^ human cortical neurons cocultured with healthy astrocytes (16). This observation, combined with the fact that *EHMT1* is most highly expressed in human astrocytes, raises the question whether EHMT1 plays a specific role in astrocyte function. In the healthy brain, astrocytes play crucial roles in neuronal network function and the homeostasis of the central nervous system, including synaptic glutamate uptake (20), synaptogenesis (21), maintenance of extracellular potassium (22), nutrient support for neurons (23), adhesion of extracellular matrix molecules (24), and many other processes (25). Consistent with such a variety of fundamental functions exerted by astrocytes to support neurons, astrocyte impairment has been shown to contribute to the pathophysiology of several neurodevelopmental disorders (26), including Fragile X syndrome (27), Rett syndrome (28), Down syndrome (29), Costello syndrome (30), and several neurodegenerative disorders (31). The converging pathogenic functions of these astrocytes often result from a gain of toxic functions, such as secretion of toxic proteins altering neuron-glia communication, or loss of homeostatic function, such as reduced glutamate uptake ability (32). Whether EHMT1 is required for the maintenance of homeostatic functions in astrocytes, and whether these astrocytic mechanisms will affect the neuronal function, is yet unknown. To improve our understanding of biological mechanisms that may underlie the brain pathology in KLEFS1 and examine the therapeutic efficacy of olanzapine, we investigated the well-characterized preclinical induced pluripotent stem cell–derived (iPSC) and ex-vivo brain slices from mouse *Ehmt1* haploinsufficiency model system by extending to mutated astrocytes.

## Results

### Decline in adaptive functioning in adolescence and young adulthood of KLEFS individuals

#### Total visits.

In this study we included 54 individuals with KLEFS1, all with confirmed aberrations of *EHMT1* (Supplemental Figure 1 and Supplemental Table 4). During the study, a total of 148 test visits took place. In 73 of these, an adapted or shorter test battery was used, due to digital modifications, SARS-CoV-2-related changes in administration, or the individual’s personal circumstances preventing completion of the full test battery. The number of visits per individual ranged from 1 (e.g., individual N24) to 7 (participant N1), with 81.5% of individuals attending at least 2 visits (Supplemental Table 6). The duration of participation, defined as the time from inclusion to the last visit, ranged from 6 months (participant B07) to 3 years and 6 months (participant N09). Additionally, 19 data points from visits of a previous study were incorporated into the dataset and analyzed (14). These retrospective data, derived from 14 different individuals (i.e., N03, N04 [seen 3 times], N06 [2 ×], N09, N12, N14, N15, N16 [2 ×], N18 [2 ×], N21, N23 [2 ×], N24 [2 ×], N39, and N41), represent scientific and clinical data of participants who have also been seen before the inclusion of the current study. The biological age at time of collection of these data is ranging from 5 years and 3 months (N23) to 36 years and 4 months (N41) (Supplemental Table 5).

#### Primary outcome: adaptive functioning (VABS/ABAS) and secondary outcome: maladaptive functioning (mini-PAS-ADD and PANSS).

**Figure 1:**
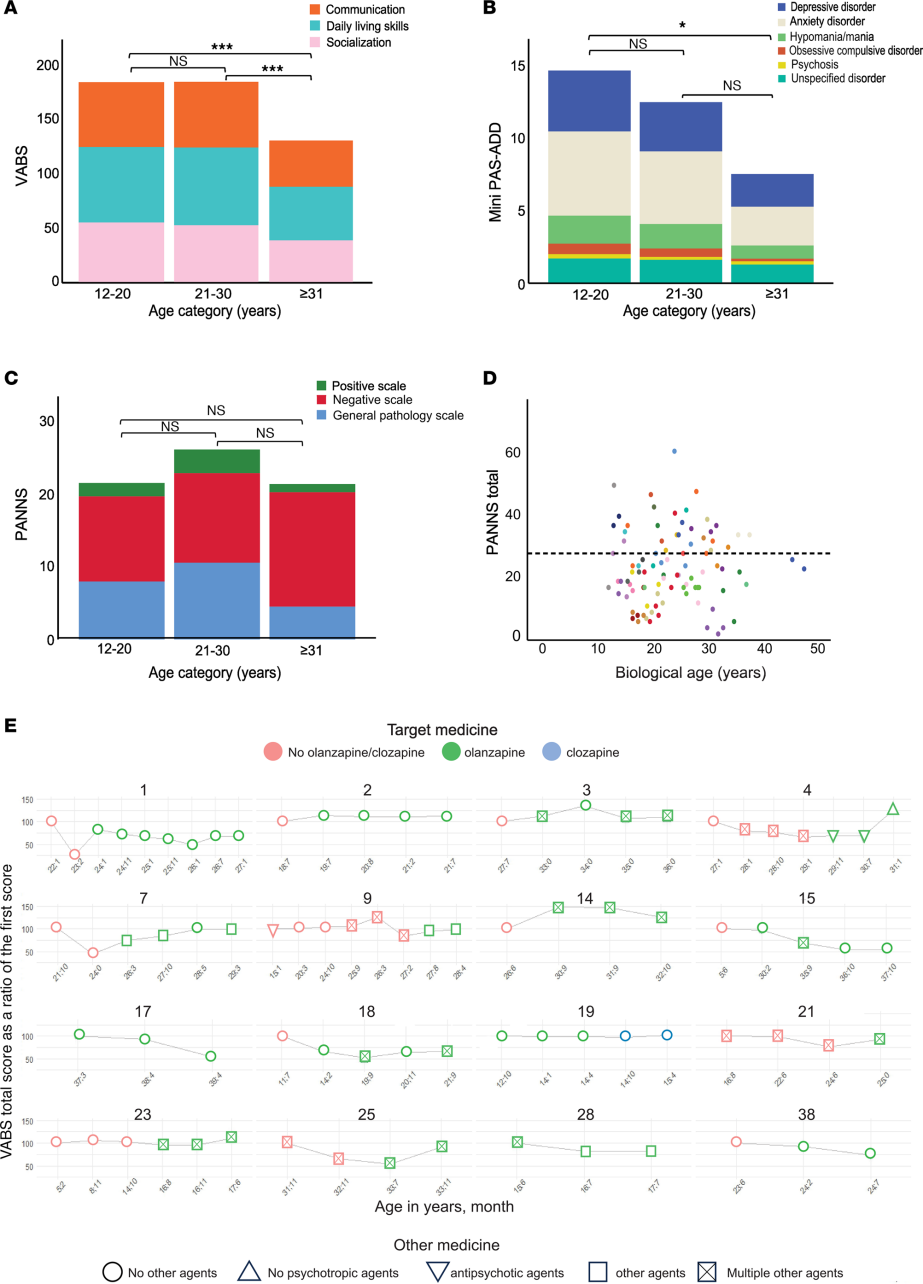
Adaptive functioning and psychopathology in our cohort of 54 individuals with KLEFS1 at different age categories. (**A**) Adaptive functioning (VABS) and its domains per age category. A total of 165 measurements were completed. *** *P* < 0.001. (**B**) Episodic psychiatric disorders measured with the mini PAS-ADD (DSM-categories). A total of 191 measurements were completed. (**C**) Total PANSS scores per domain per age category. A total of 93 measurements were completed. After the Bonferroni correction for multiple testing, differences were found between the age groups of patients aged 21–30 years and greater than or equal to 31 years on the positive, general pathology, and composite scales (significant on subscale general pathology). (**D**) Total individual PANSS scores. The horizontal line denotes the clinical cut-off of 28 points. A total of 93 measurements were completed, of which 33 measurements were on or above the cut-off point (35.5%). (**E**) Individual course over time of adaptive functioning and (olanzapine) treatment. Shows the individual course of adaptive functioning before and/or during treatment with psychiatric medication. Numbers corresponding to the Nijmegen cohort (e.g., 1= N1). The colors green and blue represent the target medicines olanzapine and clozapine, respectively. Red indicates that neither was administered. The form indicates whether other psychiatric medication was used. The category ‘other agents’ includes psychiatric medication from categories other than antipsychotics (antidepressants, lithium, antiepileptics for mood, and benzodiazepines). Treatment with olanzapine/clozapine was in accordance with Dutch guidelines regarding antipsychotic medication.

Adaptive functioning was selected as primary outcome, since this best met the needs of the participants, was in line with DSM 5 criteria and reduced the burden of participation compared with traditional neurocognitive tests (Supplemental Table 3) (8, 9, 14). Adaptive Behavior Assessment System 3rd edition (ABAS-3) scores were harmonized to align with Vineland Adaptive Behavior Scale (VABS, Dutch adaptation of Vineland-II, called Vineland-Z) scores based on our previous findings (33). Adaptive behavior scores varied widely, ranging from the lower limits of the instruments (equivalent to a developmental age of under 11 months) to the upper limits (greater than 12 years and 1 month). To facilitate age-based comparisons, individuals were grouped into 3 categories: adolescents (12–20 years), young adults (21–30 years), and adults (31+ years). We observed a decline in the level of adaptive functioning when comparing the younger age groups (12–20 years and 21–30 years) with the oldest group (aged 31 or more). Analyses were performed using a Kruskall-Wallis test. For the subdomains (communication, daily living skills, and socialization), we observed similar, statically significant, declines (Figure 1A and Supplemental Table 6).

Psychopathology was assessed using the Mini Psychiatric Assessment Schedules for Adults with Developmental Disabilities Screening (mini-PAS-ADD) (Figure 1B) and Positive And Negative Syndrome Scale (PANSS) (Figure 1, C and D). Mean scores for several diagnostic categories were higher in the younger age groups compared with the oldest group in the mini-PAS-ADD results. Additionally, differences were observed between the 21–30 and 31+ age groups on the positive, general pathology, and composite scales of the PANSS with a peak in adolescent age.

### Decline in adaptive functioning can be partially reversed by olanzapine

To determine whether there was a developmental regression in individuals, we assessed those with 3 or more adaptive measurements (*n* = 32) with no clinical suspicion for additional catatonia.

Sixteen of the participants experienced regression during the study and for this received olanzapine treatment (Figure 1E). Seven individuals clearly profited from olanzapine with recovery in functioning (numbers N1, N2, N3, N7, N9, N14, N21), 2 individuals stabilized but showed some recovery over time (N4, N23), 1 individual stabilized and showed some recovery after switch to clozapine (N19), 3 individuals stabilized for a while but continued to decline over time (N15, N17, N38), 1 individual initially declined but showed almost complete recovery 4 months later (N25), and 2 individuals further declined in functioning without any stabilization or minor recovery (N18, N28). So, in conclusion, 10 of 16 individuals did benefit in terms of improved functioning after an initial regressive period using olanzapine, and another 4 individuals stabilized at least temporarily. Extensive data points on the course of functionality that include data prior to initial regression phases were present for 7 of 10 individuals. They showed a decline in adaptive functioning during regression ranging from 25.6%– 87.2% of initial functioning, with greater range of decline in the higher functioning individuals. Recovery during olanzapine treatment in these individuals ranged from 78.4%–126% of the initial functioning. (Supplemental Figure 2). The latter indicates that the individual not only recovered to initial functioning but was able to even further improve. In 3 of 10 individuals (N2, N3, N14) data on their functioning before regression measures were lacking. Their functioning improved, respectively, 114%, 1434.9%, and 148.2% during olanzapine treatment. Dosage regimes and adverse reactions were comparable with those with idiopathic psychiatric illness (Supplemental Table 3).

### EHMT1+/– human astrocytes show increased calcium response to glutamate and altered transcriptomic profile

**Figure 2:**
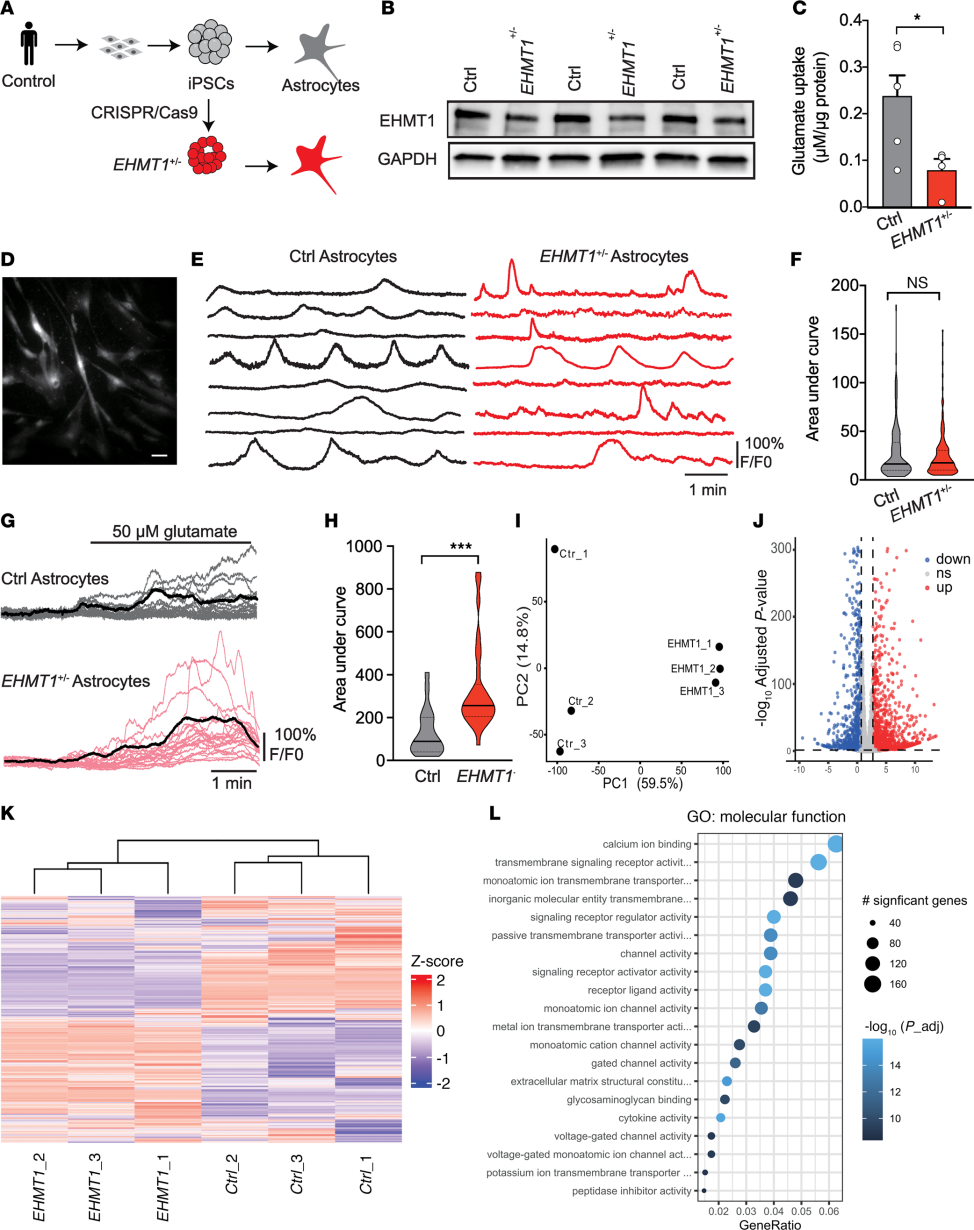
*EHMT1*^+/–^ human astrocytes show increased calcium response to glutamate and altered transcriptomic profile. (**A**) IPS cells generated through CRIPSR/cas9 used in this study. (**B**) Western blot showing the EHMT1 protein levels in control and *EHMT1^+/–^* astrocytes. (**C**) Glutamate uptake ability of control and *EHMT1^+/–^* astrocytes, *n* = 4 independent cultures (**D**) Representative image of astrocytes for calcium imaging measure using Fluo8-AM. (**E**) Representative traces of control and *EHMT1*^+/–^ astrocytes showing spontaneous calcium transients. (**F**) Quantification of AUC of spontaneous calcium transients. *n* = 139 for control and *n* = 197 for *EHMT1*^+/–^, 3 biological replicates (**G**) Representative traces of control and *EHMT1*^+/–^ astrocytes showing evoked activity upon 50 μM glutamate stimulation. Thick black trace represents the average trace. (**H**) Quantification of AUC of evoked calcium events. *n* = 21 for control and *n* = 30 for *EHMT1*^+/-^, 3 biological replicates. (**I**) Principal component analysis (PCA) of RNA-seq data from control (Ctrl) and *EHMT1*^+/–^ astrocytes, *n* = 3 independent cultures. (**J**) Volcano plots showing differentially expressed genes (DEGs) between *EHMT1*^+/–^ and control human astrocytes. Relative to the control, significantly up- or downregulated genes are shown above the black dashed line in red and blue (FDR < 0.05; log_2_(FC) > 1; log_2_(FC) < –1).(**K**) Heatmap showing relative normalized gene expression across samples. Normalized counts were log_2_-transformed and scaled per gene to Z-scores, representing relative expression levels. Both genes and samples were hierarchically clustered based on Z-scores. (**L**) GO term analysis of molecular function. Data represent means ± SEM. *P < 0.05, **P < 0.01, ***P < 0.001. Unpaired Student’s *t* test (**C**, **F** and **H**).

To investigate the cellular mechanisms underlying the beneficial effects of olanzapine, we established a cellular model system to examine its impact. Based on transcriptomic data from the Brain RNA-seq database (15) and our own RNA-seq datasets (Supplemental Figure 3A), which both indicate a higher expression level of *EHMT1* in astrocytes relative to neurons, we sought to explore its specific role in astrocytes, a cell type that has previously not been investigated in KLEFS1. We used a previously characterized *EHMT1* loss-of-function isogenic pair engineered with CRISPR/Cas9 (*EHMT1^+/–^* and *EHMT1^+/+^*) (Figure 2A) (16). All iPS cells successfully differentiated into astrocytes, using a recently described protocol (34) and expressed typical astrocytic markers, including CD44, Vimentin, GFAP, and S100B (Supplemental Figure 3, B–E). As expected, *EHMT1^+/–^* astrocytes exhibited reduced *EHMT1* expression compared with controls (Figure 2B).

A key functional property of astrocytes is their ability to support neuronal circuitry function by the uptake of extracellular glutamate. We observed that *EHMT1^+/–^* astrocytes exhibited significantly reduced glutamate uptake compared with control astrocytes (Figure 2C). Considering the crucial role of calcium signaling in astrocyte function, we next examined spontaneous cytosolic calcium dynamics in both control and *EHMT1^+/–^* astrocytes (Figure 2, D–F). While both genotypes displayed spontaneous calcium transients with no significant differences in frequency or amplitude (Figure 2F), glutamate stimulation elicited a markedly enhanced Ca²^+^ response in *EHMT1^+/–^* astrocytes compared with controls (Figure 2, G and H). These findings indicate that *EHMT1^+/–^* astrocytes exhibit altered calcium homeostasis and heightened sensitivity to glutamate-induced calcium signaling.

To investigate the molecular perturbations underlying altered astrocytic function in EHMT1-deficient astrocytes, we performed RNA sequencing on control and *EHMT1^+/–^* astrocytes. Differential expression analysis identified 1223 downregulated and 1427 significantly upregulated genes in *EHMT1^+/–^* astrocytes compared to controls (Figure 2, I and J, Supplemental Figure 4A, and Supplemental Table 7). The predominance of upregulated genes aligns with EHMT1’s known role as a transcriptional repressor (35). To obtain a broader perspective on affected pathways we performed a Gene Ontology (GO) analysis, which revealed significant enrichment in terms related to calcium ion binding, transmembrane signaling, ion transport and cytokines (Figure 2, K and L, and Supplemental Table 8).

To further gain insights into molecular underpinnings of the reduced glutamate uptake ability as well as increased calcium response to glutamate in *EHMT1*^+/–^ astrocytes, we specifically examined genes involved in these processes. Astrocytic glutamate transporters, or excitatory amino acid transporters (EAATs), play a crucial role in maintaining extracellular glutamate homeostasis. Notably, our RNA-seq analysis revealed a significant downregulation of key glutamate transporter genes (*SLC1A1*, *SLC1A3*, and *SLC1A4*) in *EHMT1*^+/–^ astrocytes (Supplemental Figure 4B). We independently validated the downregulation of these transporters via qPCR, providing a potential molecular explanation for the observed deficits in glutamate clearance (Supplemental Figure 4C).

Calcium homeostasis is regulated by a complex network of calcium channels, exchangers, binding proteins, and pumps. We identified multiple dysregulated genes associated with calcium homeostasis (Supplemental Table 8), including significant upregulation of store-operated calcium entry channel genes (*TRPC3/4/6*). Notably, some genes involved in calcium pathways, for example *RyR1* and *CACNA1H,* exhibited dramatic increases of 626-fold and 53-fold, respectively, suggesting a major contribution to calcium signaling abnormalities in *EHMT1^+/–^* astrocytes. Additionally, several calcium-binding protein genes, such as *CAMK2N1* and *S100B*, displayed altered expression (Supplemental Tables 7 and 8). Collectively, our findings indicate profound transcriptomic dysregulation in *EHMT1^+/–^* astrocytes, implicating disruptions in glutamate transport, calcium signaling, and broader cellular processes. Of interest, although the transcriptome of *EHMT1^+/–^* neurons (grown on control astrocytes) was more moderately affected (Supplemental Figure 4, D–H), GO term analysis revealed a similar enrichment in calcium ion binding (Supplemental Figure 4H and Supplemental Table 7).

### Olanzapine rescues the neuronal network hyperactivity in EHMT1+/– astrocyte-neuron cocultures

**Figure 3:**
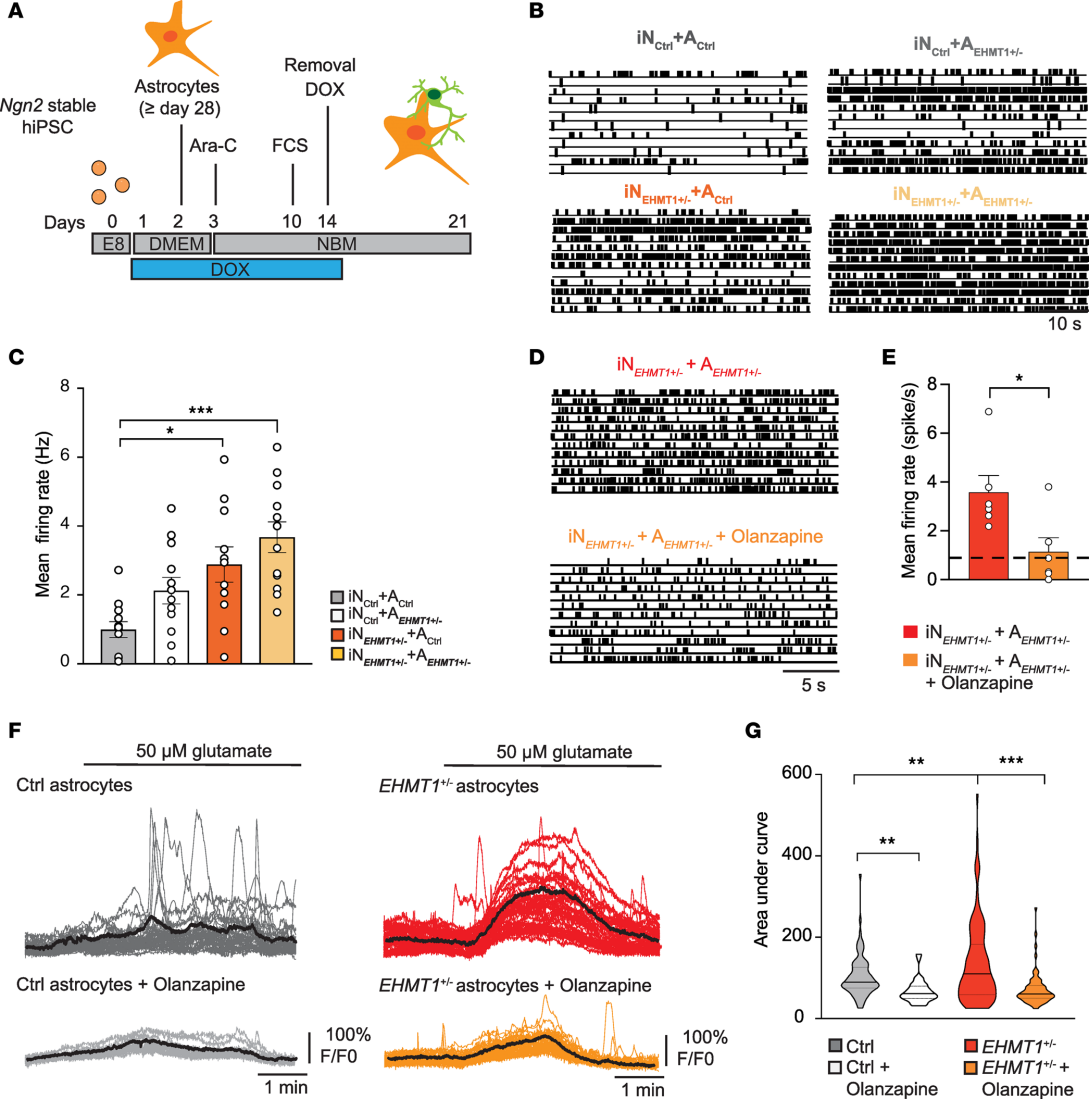
*EHMT1*^+/–^ human astrocytes lead to neuronal network hyperactivity in *EHMT1*^+/–^ neurons, which can be rescued by olanzapine. (**A**) Schematic overview of human astrocyte and human neuron cocultures. (**B**) Representative raster plot and (**C**) quantification of mean firing rate exhibited by control or *EHMT1*^+/–^ astrocyte coculture with control or *EHMT1*^+/–^ human neurons on MEA at DIV21. iN_Ctrl_+A_Ctrl_ *n* = 12, iN_Ctrl_+A_EHMT1+/–_ *n* = 12, iN_EHMT1+/–_+A_Ctrl_ *n* = 11 and iN_EHMT1+/–_+A_EHMT1+/–_ *n* = 12. Data represent means ± SEM. *P < 0.05, **P < 0.01, ***P < 0.001. One-way ANOVA test and post hoc Bonferroni correction (**D**) Representative raster plots and (**E**) quantification of mean firing rate exhibited by *EHMT1*^+/–^ astrocyte-neuron cocultures on MEAs treated with 10 μM olanzapine for 1 week. Dashed line represents mean of untreated control cells, *n* = 6. Data represent means ± SEM. *P < 0.05, **P < 0.01, ***P < 0.001. Unpaired Student’s *t* test (**E**). (**F**) Representative traces and (**G**) quantification of calcium wave from control and *EHMT1*^+/–^ astrocytes treated with/without olanzapine upon 50 μM glutamate stimulation. Control *n* = 94, Control + olanzapine *n* = 54, *EHMT1*^+/–^ *n* = 104, *EHMT1*^+/–^ + olanzapine *n* = 81 Data represent means ± SEM. *P < 0.05, **P < 0.01, ***P < 0.001. One-way ANOVA test and post hoc Bonferroni correction (**G**).

To investigate whether astrocytes contribute to neuronal network dysfunction in KLEFS1, we cocultured human control or *EHMT1*^+/–^ iPSC-derived *Ngn2*-induced glutamatergic neurons (iN) together with human astrocytes in an all-human model system. This allowed us to generate networks in which neurons and astrocytes of different genotypes can be cocultured in different combinations (i.e., iN_Ctrl_+A_Ctrl_, iN_Ctrl_+A_Ehmt1+/–_, iN_EHMT1+/–_+A_Ctrl_ and iN_EHMT1+/–_+A_EHMT1+/–_, Figure 3A). After 3 weeks of development in vitro, we measured the neuronal activity on MEAs (Figure 3B). iN_EHMT1+/–_+A_EHMT1+/–_ networks showed a significant increase in their global activity level compared with iN_Ctrl_+A_Ctrl_ networks (Figure 3C). Specifically, we found that the mean firing rate (MFR) of *EHMT1*^+/–^ neurons was exacerbated when grown on *EHMT1*^+/–^ astrocytes (iN_EHMT1+/–_+A_EHMT1+/–_, yellow) compared with culturing *EHMT1*^+/–^ neurons on control astrocytes (iN_EHMT1+/–_+A_Ctrl_, orange). Importantly, we found no difference in the density of astrocytes between the different conditions, nor was the ratio between astrocytes (GFAP) and neurons (MAP2) different between conditions (Supplemental Figure 3F).

To corroborate these data, we also generated networks composed of human iN and mouse cortical astrocytes isolated from control or *Ehmt1*^+/–^ mice, as rodent astrocytes are considered the gold standard in this type of in vitro models (Supplemental Figure 5, A and B). Networks consisting of mouse astrocytes showed more distinct synchronization of spontaneous network activity — represented by network bursts — after 3 weeks, compared with all-human cultures. We first recapitulated the glutamatergic neuron–specific network phenotype in iN_EHMT+/–_+ mouseA_Ctrl_, as we described before (16) (orange), which included a lower network burst rate and a longer burst duration compared with iN_Ctrl_+mouseA_Ctrl_ networks (dark grey) (Supplemental Figure 5, C–F). Similar to the all-human cultures, we found that the network phenotype of *EHMT1*^+/–^ neurons was exacerbated when grown on mouse *Ehmt1*^+/–^ astrocytes (iN_EHMT+/–_+mouseA_Ehmt1+/–_, yellow) compared with culturing *EHMT1*^+/–^ neurons on control astrocytes (Supplemental Figure 5C). Indeed, iN_EHMT+/–_+mouseA_Ehmt1+/–_ networks showed a significant increase in MFR compared with iN_EHMT+/–_+mouseA_Ctrl_ networks (Supplemental Figure 5D). Together, these results confirm that astrocytes significantly contribute to neuronal network dysfunction in KLEFS1. Notably, when control neurons were grown with *EHMT1*-deficient astrocytes (iN_Ctrl_+mouseA_Ehmt1+/–_), we did not observe any significant differences in neuronal network activity compared with iN_Ctrl_+mouseA_Ctrl_ networks. These results suggest that *EHMT1* deficiency in astrocytes alone is not sufficient to induce a major neuronal network phenotype. Moreover, these findings suggest that *EHMT1*^+/–^ neurons are more sensitive than control neurons to the presence of EHMT1-deficient astrocytes, resulting in the observed cumulative effect on the network phenotype. Consistently, when measuring spontaneous excitatory postsynaptic currents (sEPSCs) using whole-cell patch-clamp recordings, we found that mouse *Ehmt1*^+/–^ astrocytes aggravated the single-cell phenotype of *EHMT1*^+/–^ neurons by means of an increased sEPSC frequency but not amplitude (Supplemental Figure 5, G–I).

Having established an all-human model system exhibiting a clear *EHMT1* haploinsufficiency phenotype, we next investigated whether olanzapine could rescue the associated neuronal hyperactivity. To this end, we treated *EHMT1^+/–^* astrocyte-neuron cocultures with olanzapine for a week and compared them with untreated controls. olanzapine effectively reduced overall network activity to levels comparable with those of untreated control networks (Figure 3, D and E). This effect seemed to be specific for olanzapine, as other antipsychotics such as quetiapine and aripiprazole did not reduce overall network activity in EHMT1^+/–^ astrocyte-neuron cocultures (Supplemental Figure 6).

Given our previous results showing that *EHMT1* haploinsufficient astrocytes exhibited enhanced calcium response to glutamate stimulation and considering evidence that astrocytic calcium activity can influence neuronal excitability along with the previous reported impact of olanzapine on calcium signaling (36), we tested whether olanzapine could also normalize calcium signaling in *EHMT1^+/–^* astrocytes. Indeed, olanzapine treatment reduced the heightened calcium response to glutamate stimulation in *EHMT1^+/–^* astrocytes (Figure 3, F and G). These findings suggest that olanzapine can mitigate the hyperactive neuronal network phenotype associated with EHMT1 deficiency, potentially through its stabilizing effect on aberrant astrocytic calcium signaling.

To corroborate our results in an independent isogenic KS patient line, we generated iNeurons and astrocytes from previously characterized isogenic iPSCs obtained from a female who has a mosaic microdeletion on chromosome 9q34 (233 kb), including *EHMT1 (KS_mos_ and C_mos,_* Supplemental Figure 6, A and B) (16). KS_mos_ neuronal networks were hyperactive compared with control C_mos_ networks (Supplemental Figure 7, C and D). This was accompanied by increased calcium response to glutamate. Importantly, these phenotypes could be rescued by treating the cells with olanzapine (Supplemental Figure 7E). Together, these data indicate a more general mechanism at play in KLEFS1.

Because EHMT1 is also expressed in other cell types, including dopaminergic and serotonergic neurons, and olanzapine is also known to exert some of its effects by modulating dopaminergic (D2) and serotonergic (5-HT2A) receptors (37), we explored the role of EHMT1 in these cells. To this end, we generated human iPSC-derived dopaminergic and serotonergic neurons (Supplemental Figure 8) and grew them on MEAs to measure changes in neuronal network activity. We performed a detailed analysis of neuronal network properties during development, but we did not observe any neuronal network phenotype between control and *EHMT1*-haploinsufficient dopaminergic or serotonergic neurons (Supplemental Figure 8). This suggests that these cell types are not directly affected by *EHMT1* haploinsufficiency.

### EHMT1+/– human astrocytes show increased S100B, which can be reduced by olanzapine treatment

We next sought to understand the underlying mechanisms by which olanzapine rescues abnormal calcium activity in *EHMT1^+/–^* astrocytes and attenuates hyperactive neuronal activity. Examining our astrocytic transcriptomic data, we were particularly interested in S100B, a key regulator of calcium wave propagation. Our RNA-seq data revealed an increased expression of *S100B* mRNA in *EHMT1^+/–^* astrocytes. Notably, previous studies have reported that olanzapine can reduce S100B levels (38). Based on this, we hypothesized that the elevated S100B expression in *EHMT1^+/–^* astrocytes could contribute to the observed calcium dysregulation and hyperactive neuronal network phenotype, and that olanzapine potentially exerts its rescue effect by downregulating S100B expression.

**Figure 4:**
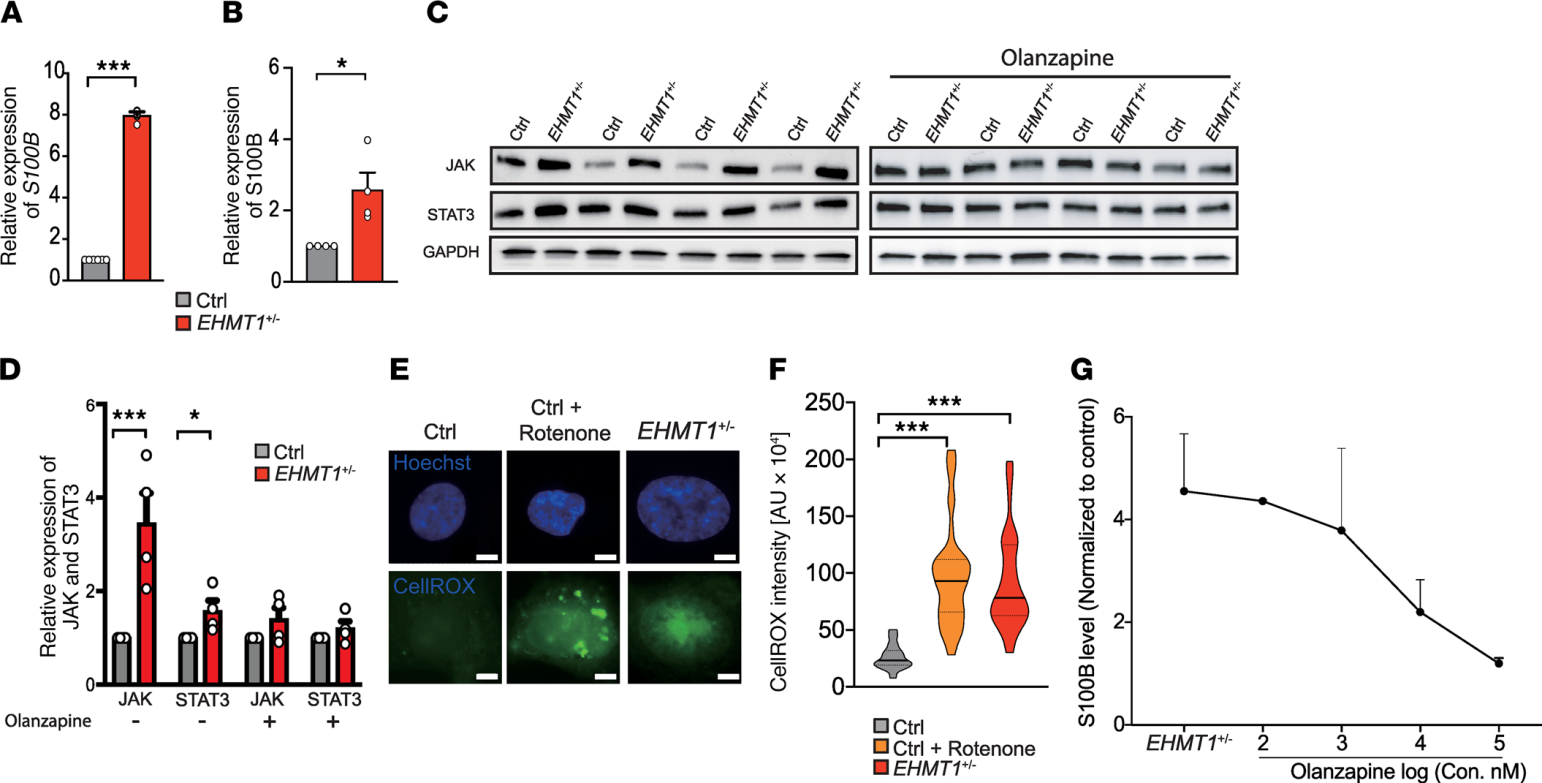
*EHMT1*^+/–^ human astrocytes show increased S100B, and the level of S100B can be reduced by olanzapine treatment. (**A**) Relative expression of *S100B* in control and *EHMT1*^+/–^ astrocytes using qPCR. Control *n* = 6, *EHMT1*^+/–^ *n* = 6. (**B**) Relative protein level of S100B in the culture medium of control and *EHMT1*^+/–^ astrocytes using ELISA. Control *n* = 4, *EHMT1*^+/–^ *n* = 4. (**C**) Western blot showing the JAK/STAT3 protein levels in astrocytes in the absence or presence of olanzapine (**D**) quantification of Western Blot, normalized to control. Control *n* = 4, *EHMT1*^+/–^ *n* = 4. (**E**) Representative images and (**F**) quantification of CellROX fluorescent intensity in control, control + Rotenone and *EHMT1*^+/–^ astrocytes. Control *n* = 34, Control + 100 nM Rotenone *n* = 34, *EHMT1*^+/–^ *n* = 41. (**G**) olanzapine (100 nM, 1 μM, 10 μM, 100 μM) treatment on *EHMT1^+/–^* astrocytes for 1 week could reduce S100B level in the culture medium in a dose-dependent manner as tested by ELISA. *n* = 3. Data represent means ± SEM. *P < 0.05, **P < 0.01, ***P < 0.001. One-way ANOVA test and post hoc Bonferroni correction (**F**) and unpaired Student’s *t* test (**A**, **B**, and **D**).

To test this hypothesis, we first confirmed, in independent experiments, that *EHMT1* haploinsufficiency leads to increased *S100B* transcript and extracellular protein expression from human astrocytes. We performed both qPCR and ELISA for control and *EHMT1*^+/–^ astrocytes at DIV 28. The results showed that, at both mRNA and protein levels, *EHMT1* haploinsufficiency resulted in a significant increase of S100B (Figure 4, A and B). Further, using CUT&RUN-qPCR we measured reduced H3K9me2 occupancy at *S100B* promoter in *EHMT1*^+/–^ astrocytes (Supplemental Figure 9A). In addition, we observed that 4 out of 8 CpG sites showed prominently decreased DNA methylation in *EHMT1*^+/–^ astrocytes compared with control astrocytes (Supplemental Figure 9B). These results suggest that *EHMT1* deficiency directly or indirectly results in reduced H3K9me2 and DNA methylation at the *S100B* promoter in astrocytes, possibly responsible for the increased expression of *S100B*.

At nanomolar concentrations, S100B has been shown to activate the Ras/ERK/NF-kB pathway to promote neurotrophic functions (39, 40). However, when released at high concentrations (micromolar range) (41), S100B is suggested to act as a Damage-Associated Molecular Pattern (DAMP) molecule that triggers several stress response pathways (42). These include, among others, the JNK/p38 and the Janus Kinase (JAK)/signal transducer and activator of transcription (STAT) pathways, which are known to serve as downstream effectors potentially relevant to reactive oxygen species (ROS) production and reactive gliosis (42–45). This raised the question whether *EHMT1*^+/–^ astrocytes showed aberrant activation of JAK/STAT signaling and accumulated higher ROS levels. To test this, we performed immunoblotting of JAK and STAT3. *EHMT1*^+/–^ astrocytes showed significantly higher levels of total JAK and STAT3 (Figure 4, C and D). Strikingly, the higher levels of total JAK and STAT3 were abolished when cells were pretreated with olanzapine (Figure 4, C and D). Next, we assessed ROS levels using CellROX Green in *EHMT1*^+/–^ astrocytes. As a positive control, we included control astrocytes treated with Rotenone, a mitochondrial complex one inhibitor, to induce oxidative stress (46). *EHMT1*^+/–^ human astrocytes produced significantly higher ROS than control astrocytes (Figure 4, E and F). Consistent with the data collected from human astrocytes, *Ehmt1*^+/–^ mouse astrocytes showed a similar increase in *S100b* (Supplemental Figure 10, A, C–-E, and H–J) and produced more ROS than control astrocytes in primary cultures (Supplemental Figure 10B). In line with these in vitro data, we also observed increased S100B, GFAP expression, and GFAP area in brain slices of the visual cortex and hippocampus of *Ehmt1*^+/–^ mice, suggesting increased astrocyte reactivity and signs of neuroinflammation ex vivo (Supplemental Figure 10, C, F–H, K, and L). This further emphasizes the cross-species changes in S100B expression and increased oxidative stress in astrocytes with EHMT1 deficiency.

Treatment of olanzapine to individuals with schizophrenia (SCZ) for 2 weeks has been shown to significantly reduce plasma S100B level (38). Considering that astrocytes are the main cell type that could secrete S100B, we further explored whether, in our human astrocyte model, olanzapine could reduce S100B expression. To test this, we treated *EHMT1*^+/–^ astrocytes with different doses of olanzapine for 1 week, after which we measured S100B protein level. We found that olanzapine reduced the extracellular S100B level in a dose-dependent manner (Figure 4G). Taken together, this indicates that *EHMT1*^+/–^ astrocytes display an upregulation in S100B, which can be attenuated by the administration of olanzapine.

S100B inhibition in *EHMT1*^+/–^ astrocytes rescues the neuronal network hyperactivity in *EHMT1*^+/–^ astrocyte-neuron cocultures.

**Figure 5:**
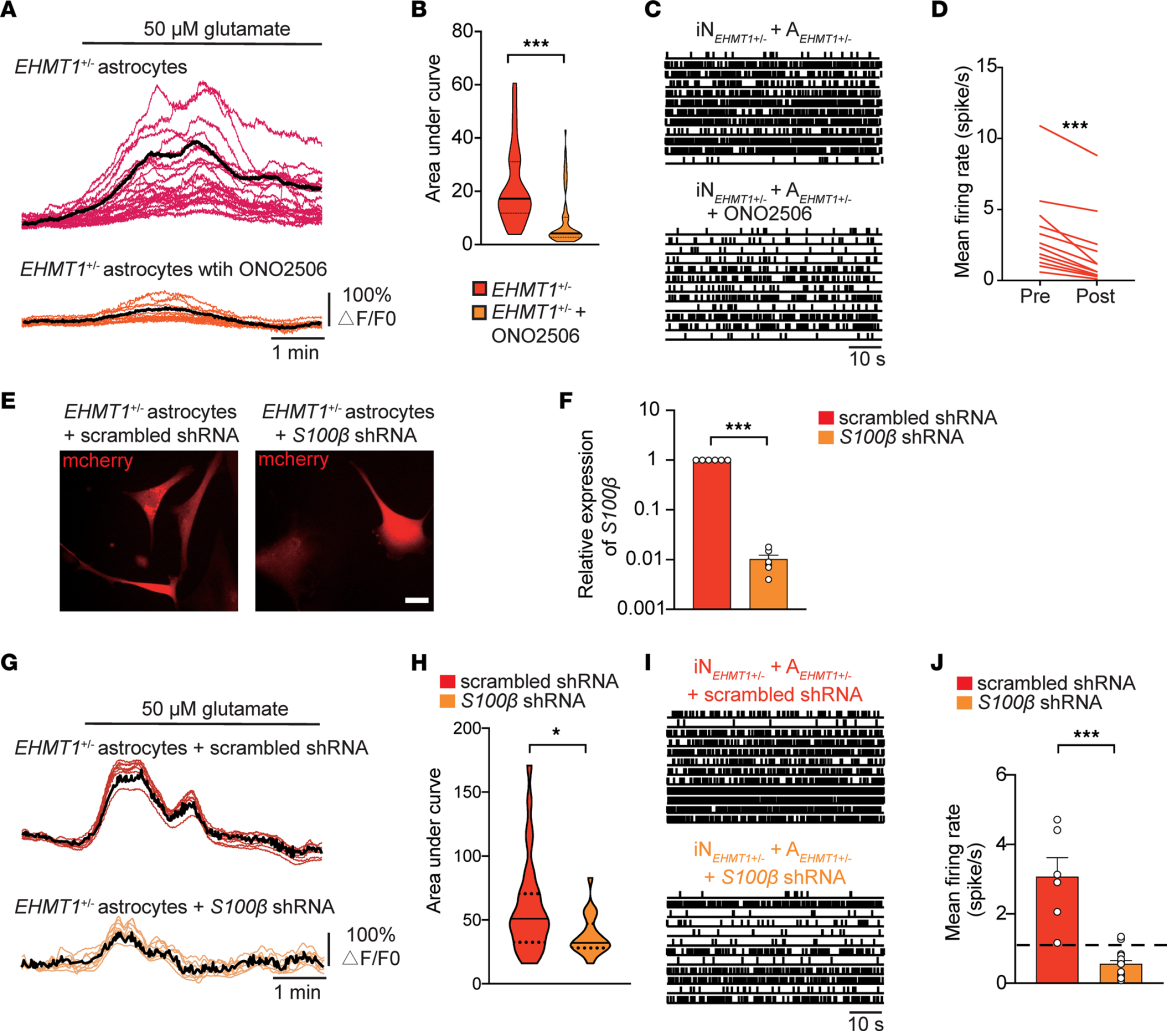
Pharmacological inhibition and genetic knockdown of *S100B* in *EHMT1*^+/–^ astrocytes rescue the neuronal network hyperactivity in *EHMT1*^+/–^ astrocyte-neuron cocultures. (**A**) Representative traces and (**B**) quantification of *EHMT1*^+/–^ astrocytes with ONO2506 showing reduced evoked activity upon 50 μM glutamate stimulation. *n* = 57 for *EHMT1*^+/–^ and *n* = 63 for *EHMT1*^+/–^ with ONO2506. (**C**) Representative raster plots and (**D**) quantification of mean firing rate exhibited by *EHMT1*^+/–^ astrocytes cocultured with *EHMT1*^+/–^ neurons on MEAs at DIV21 after 24-hour treatment of 100 μM ONO2506. *n* = 12. (**E**) *EHMT1*^+/–^ astrocytes infected lentivirus expressing mcherry and scrambled or *S100B* shRNA. (**F**) Relative expression of mRNA *S100B* in *EHMT1*^+/–^ astrocytes infected with *S100B* shRNA compared with astrocytes transfected with scrambled shRNA. *n* = 6 for each group. (**G**) Representative traces and (**H**) quantification of calcium wave from *EHMT1*^+/–^ astrocytes transfected with scrambled shRNA and *S100B* shRNA upon 50 μM glutamate stimulation. *n* = 29 for *EHMT1*^+/–^ + scrambled shRNA and *n* = 21 for *EHMT1*^+/–^ + *S100B* shRNA. (**I**) Representative raster plots and (**J**) quantification of mean firing rate exhibited by *EHMT1*^+/–^ neurons cocultured with *EHMT1*^+/–^ astrocytes transfected with scrambled shRNA or *S100B* shRNA on MEAs. Dashed line represents mean of untreated control cells, *n* = 6 for scrambled shRNA. *n* = 15 for *S100B* shRNA. Data represent means ± SEM. *P < 0.05, **P < 0.01, ***P < 0.001. Unpaired Student’s *t* test (**B**, **F**, **H**, and **J**) and paired Student’s *t* test (**D**).

S100B has been reported as one of the important regulators in calcium signaling (44). We next investigated whether reducing S100B expression would be sufficient to revert the increased Ca^2+^ responses and neuronal network hyperactivity phenotypes. To pharmacologically reduce S100B expression, we treated *EHMT1*^+/–^ astrocytes with the *S100B* synthesis inhibitor, ONO2506, for 24 hours. We observed that ONO2506 significantly reduced the increased calcium response to glutamate of *EHMT1*^+/–^ astrocytes (Figure 5, A and B). Considering that calcium signaling in astrocytes could potentially influence neuronal excitability, we next tested whether, by pharmacologically inhibiting S100B, the hyperactive network phenotype in iN_EHMT1+/–_+A_EHMT1+/–_ could be rescued. After 24 hours ONO2506 treatment (100 μM), we found that the global activity of iN_EHMT1+/–_+A_EHMT1+/–_ cultures was significantly reduced (Figure 5, C and D). Next to pharmacological inhibition, we also used short hairpin RNA (shRNA) to genetically knock down *S100B* (Figure 5E). qPCR data confirmed that *S100B* shRNA significantly decreased *S100B* expression (Figure 5F). Consistently, both increased glutamate-evoked calcium response in *EHMT1*^+/–^ astrocytes and the hyperactive network phenotype in astrocyte-neuron cocultures were normalized to control levels (Figure 5, G–J). Thus, we conclude that the hyperactivity of *EHMT1*^+/–^ network, at least partially, is caused by increased S100B from *EHMT1*^+/–^ astrocytes; and olanzapine could rescue the *EHMT1*^+/–^ network phenotype via reducing S100B. Collectively, these data offers a new mechanistic perspective on how olanzapine can impact *EHMT1* haploinsufficient neuronal networks.

## Discussion

Developmental regression poses a substantial challenge in NDDs, particularly in genetic syndromes such as KLEFS1, where individuals experience major loss of function, profoundly impacting quality of life. In this study, we systematically characterized the course of developmental regression and treatment effects in an international cohort of individuals with KLEFS1 by a sequential design study set up. Our findings confirm a major decline in adaptive functioning with age, particularly after approximately 16 years, often accompanied by episodic psychiatric disorders. The higher prevalence of mood disorders, anxiety, OCD, and psychotic disorders in adolescence and early adulthood, followed by apathy in later stages, aligns with previous case reports and suggests an evolving disease trajectory (Supplemental Table 2). Our treatment strategy revealed that 10 of 16 individuals with such decline benefited from olanzapine, varying from significant and stable to mild, temporary improvement of adaptive functioning. It remains unclear whether the observed decline during treatment in 2 individuals truly reflects a continuous and progressive deterioration, or whether intermittent fluctuations in functioning may have occurred. Further research involving assessments at shorter intervals is warranted to obtain a better understanding of these patterns.

We identified developmental regression as primarily presenting with disorganized or catatonic psychotic symptoms, with no individuals meeting DSM-5 criteria for catatonia before treatment. This could be attributed to early systematic screening, but literature suggests that full-blown catatonia is not uncommon in KLEFS1 (10, 47). Given the high morbidity and mortality risk associated with catatonia, early psychiatric consultation — integrated with somatic diagnostics — is crucial when KLEFS1 patients exhibit sudden sleep disturbances, functional decline, and disorganized behavior. Prior to initiating olanzapine treatment, somatic causes of regression were ruled out, and no severe medical conditions were identified as primary drivers of psychopathology.

### S100B as a potential biomarker

Increased S100B levels have been reported in patients with insomnia, and S100B levels increase in healthy individuals upon sleep deprivation (48–50). Of note, patients with KLEFS1 did not respond to typical antipsychotic drugs but did respond well to olanzapine and aripiprazole (9). In mouse striatum, chronic olanzapine exposure in medium spiny neurons and microglia showed reduced S100B expression (51). In addition, olanzapine has been shown to reduce S100B levels in first-episode patients with psychotic SCZ (38). In agreement with this, we observed that olanzapine could reduce S100B level in *EHMT1*^+/–^ astrocytes in a dose-dependent manner, counteract the upregulated JAK/STAT pathway, and rescue the hyperactive network phenotype. Pharmacological inhibition or genetic knockdown of S100B similarly restored network function. This data suggests that S100B might be one of the downstream targets of the therapeutic effect of olanzapine.

S100B upregulation has been implicated in numerous neurological and psychiatric conditions, including schizophrenia (52), bipolar disorder (53), and major depressive disorder (54). Given its detectability in cerebrospinal fluid, blood, urine, saliva, and amniotic fluid, S100B could potentially serve as a biomarker for disease severity in KLEFS1. Elevated S100B levels in SCZ have been correlated with PANSS negative symptom scores and have been shown to decrease following antipsychotic treatment (55). Measuring S100B levels in patients with KLEFS1 before and after regression periods could provide critical insights into its role as a biomarker and its potential utility in monitoring disease progression and treatment response.

Mechanistically we found that olanzapine could reduce S100B expression in *EHMT1*^+/–^ expression, which may stem from multiple mechanisms. First, olanzapine has previously been shown to modulate histone modifications (H3K4me and H3K9me), suggesting an epigenetic regulation of S100B expression (56). Second, olanzapine alters DNA methylation in the hippocampus, with increased methylation observed in the *S100B* promoter, potentially suppressing its expression (57). Third, *EHMT1*^+/–^ astrocytes exhibit increased ROS production and present a reactive phenotype in vitro and ex vivo. Reactive astrocytes have been shown to release multiple neuroinflammatory cytokines such as IL-1β and IL-6 (58–60), both of which stimulate the secretion of S100B (61). Since olanzapine is able to reduce inflammatory cytokines, including IL-1β and IL-6 (62–64), one possibility is that olanzapine reduces S100B indirectly by dampening astrocyte-mediated neuroinflammation. Our data showing rescued effect of olanzapine on the upregulated JAK/STAT pathway are in line with this and suggest that the mode of action of olanzapine in KLEFS1 might be through its antiinflammatory effect. Further studies would be need to investigate these different hypotheses and to investigate other psychotropics involved in a positive course of KLEFS1.

Together, a more complete picture of the pathophysiology of KLEFS1 is emerging, in which *EHMT1* deficiency affects neuron and astrocyte-specific pathways, leading to general hyperexcitability (Supplemental Figure 11).

### Limitations of the study

Natural history studies in rare disorders are challenging, especially in vulnerable patient populations, as we present here. Though we obtained a relatively large cohort size, the findings need to be extended in future cohorts, both in terms of number of individuals and in terms of duration of treatment, including the long-term effects. Furthermore, there are several variables that need to be considered, such as the individual causes of the decline, treatment, and observer variation, which we tried to minimize by performing a strict study protocol with the same team members for the repeated measures. Regarding the preclinical model, this study focused on the under-studied and increasingly appreciated role of astrocytes in neurodevelopmental disorders. However, previous research has established that EHMT1 also exerts cell-type–specific functions in several other lineages, including neurons (16) and microglia (19).

### Conclusions

Here, we present a unique complementary study on the clinical longitudinal course and treatment intervention of severe developmental regression in KLEFS1, paralleled by extensive mechanistic studies in a relevant preclinical model of *EHMT1*-deficient brain cells. These findings can further tune disease management and treatment directions.

Our study underscores the urgent need for longitudinal monitoring in KLEFS1 and highlights the potential of olanzapine in mitigating functional decline in the context of disorganized behavior and catatonic symptoms. We recommend assessing the adaptive functioning annually, at least during puberty, and biannually when the functioning is stable in adulthood to trace the more gradual presentations in individuals who have been detected as having KLEFS1. In case of a (suspected) serious psychiatric illness, the following rating scales can be used to map the clinical course: PANSS (psychosis), mini PAS-ADD (mood disorders [uni- and bipolar], anxiety disorders, obsessive compulsive disorders, undefined disorders) and the Bush-Francis rating scale (catatonia) (65–67).

Additionally, our findings position S100B as a promising biomarker and therapeutic target, offering a path toward more precise interventions. Future work should explore the molecular mechanisms linking EHMT1 deficiency, astrocyte dysfunction, and S100B regulation, to refine treatment strategies for KLEFS1 and related disorders.

## Methods

### Sex as a biological variable

For the clinical study both males and females were studied. For the animal and iPSC studies both males and females were included and no differences were observed.

### Patient information and iPSC line generation

In this study we used two isogenic lines: an isogenic CRISPR/Cas9 generated iPSC pair WT and *EHMT1*^+/–^, and iPSCs obtained from a female who has a mosaic microdeletion on chromosome 9q34 (233 kb) including *EHMT1*. Both isogenic pairs have been previously characterized (16). iPSCs were always cultured in Essential 8 Flex Medium Kit (Gibco, A2858501) supplemented with Primocin (0.1 μg/ml, Invivogen, ant-pm-2) and low Puromycin (0.5 μg/ml, Sigma-Aldrich, P9620) and G418 (50 μg/ml, Sigma-Aldrich, G8168) concentrations on Matrigel coated plates (Corning, 356231) at 37˚C/5% CO2. Medium was refreshed every 2-3 days and cells were passaged twice per week using ReLeSR (StemCell Technologies, 100-0483). All iPSCs were regularly tested for mycoplasma infection and consistently found mycoplasma free. All iPSCs were genomically assessed with iCS-digital PSC-24 probes service (Stem Genomics) after rtTA/Ngn2 stabilization. All karyotypically normal cell lines were used within 10 passages of genomic integrity assessment.

### Animals

For the experiments presented in this study, mice heterozygous for a targeted loss-of-function variant in the *Ehmt1* gene *(Ehmt1*^+/−^ mice) and their control littermates on C57BL/6 background were used. All experiments on animals were carried out in accordance with the approved animal care and use guidelines of the Animal Care Committee, Radboud University Medical Centre, the Netherlands, (RU- DEC-2011-021, protocol number: 77073).

### Isolation and culturing of rodent astrocytes

Mouse astrocytes were isolated from P2 Wild type and *Ehmt1*^+/–^ mice in a Petri dish containing Leibovitz’s L-15 Medium (Gibco, 11415049) supplemented with B-27 supplement (Gibco, 17504044). We dissociated these cortices using 4.5 mL HBSS (Gibco, 14185045) supplemented with 500 μl of 2.5% Trypsin (Gibco, 15090046) 37˚C for 25 to 35 minutes while flicking the tube every 5 minutes. We inactivated the trypsin solution by adding culture medium (filter sterilized DMEM, high glucose, GlutaMAX Supplement, pyruvate (Gibco, 31966021) with 10% FBS (Sigma-Aldrich, F7524) and Pen/Strep (Sigma-Aldrich, P4333)) in a ratio of 1:1, followed by vigorous shaking of the tube. These cells were filtered through a 70 μm cell strainer. We spun down the total cell volume and resuspended the cells in culture medium and cultured at 37˚C/5% CO2. We exchanged 100% of the culture medium two days after plating and expanded the cells for approximately 7 to 10 days while refreshing the medium every three days. After expansion, we removed non-astrocytic cells by placing the flask on a shaker platform for 7 hours at 240 rpm and discarded the suspended cells. Hereafter, we refreshed 100% of the medium every three days.

### Neuronal differentiation

IPSCs were differentiated into glutamatergic neurons by overexpressing mouse Neurogenin 2 (*Ngn2*) upon doxycycline treatment according to a previously published protocol (16). IPSCs were detached using Accutase (Sigma-Aldrich; A6946) and resuspended in E8 basal medium (Thermo Fisher Scientific) supplemented with primocin (0.1 μg/ml, Invivogen), RevitaCell (Thermo Fisher Scientific), and doxycycline (4 μg/ml) to induce *Ngn2* expression. Cells were plated onto MEAs (600 cells/mm2) or glass nitric- acid treated coverslips (100 cells/mm2) pre-coated with Poly-L-Ornithine (50 μg/mL, Sigma) for 3 hours at 37 degrees after which plates were washed and incubated with mouse laminin (20 μg/mL, Sigma) overnight at 4°C). At days in vitro (DIV0 1 the medium was changed to DMEM-F12 (Thermo Fisher Scientific) supplemented with N2 (1:100, Thermo Fisher Scientific), non-essential amino acids (1:100, Sigma), primocin (0.1 μg/ml, Invivogen), NT3 (10 ng/ml), BDNF (10 ng/ml), and doxycycline (4μg/ml). To support neuronal maturation, freshly prepared mouse or human astrocytes were added to the culture in a 1:1 ratio two days after plating. At DIV 3 the medium was changed to Neurobasal medium (Thermo Fisher Scientific) supplemented with B-27 (Thermo Fisher Scientific), glutaMAX (Thermo Fisher Scientific), primocin, NT3, BDNF, and doxycycline. Cytosine b- D- arabinofuranoside (Ara-C) (2μM; Sigma) was added once to remove any proliferating cell from the culture. From DIV 6 onwards half of the medium was refreshed every other day. From DIV 10 onwards, the medium was supplemented with 2.5% FBS (Sigma) to support astrocyte viability. Neuronal cultures were kept through the whole differentiation process at 37°C/5%CO2.

### Human astrocyte differentiation

IPSCs were differentiated into astrocytes as described before (34). In brief, iPSCs were detached from feeder cells and suspended colonies were transferred to non-adherent plates on a shaker to promote the formation of EBs. These suspended colonies were cultured on a shaker to promote the formation of embryonic bodies (EBs). EBs were cultured for 2 days in knockout serum based medium (Advanced DMEM/F12 (Thermo Fisher cat. no. 12634028) + 20% Knockout serum (Thermo Fisher cat. no. 10828028), 1% L-glutamine (Thermo Fisher cat. no. 25030024), 1% penicillin-streptomycin (P/S; Thermo Fisher cat. no. 15140122), 1% Nonessential amino acids (Thermo Fisher cat. no. 11140035), 0.0007% 2-mercaptoethanol (Sigma cat. no. M3148) and 10 ng/ml FGF2 (Merck cat. no. GF003AF)), after which they were switched to neural induction medium (Advanced DMEM/F12, 2 μg/ml heparin (Sigma cat. no. H3149), 1% N2 supplement (Thermo Fisher cat. no. 17502048), 1% P/S). After 7 days of differentiation, EBs were gently dissociated and plated onto laminin-coated dishes (Sigma, L2020, 20μg/ml) in neural induction medium for 8 days, resulting in a population of pre-NPCs. Pre-NPCs were replated at day 15 into NPC medium (Advanced DMEM/F12 + 1% P/S + 2% N2 +2% B27-retinoic acid (Thermo Fisher Scientific cat. no. 12587-010) + 1μg/mL mouse Laminin+ 20 ng/ml FGF basic (Merck Millipore cat. no. GF003AF) to promote selection and proliferation of precursor cells. Pure populations of neural stem cells were isolated by FACS sorting the NPCs for the markers CD184+/CD271−/CD44−/CD24+ as described previously (68). From this point on NPCs were frozen down. NPCs were thawed and maintained for limited passages (max 4) in NPC medium as described above. NPCs medium was changed every other day. NPCs were passaged 1:4 once a week upon confluence using Accutase solution and replated on laminin-coated plates (20μg/ml).

### Immunocytochemistry

At DIV 21 neurons were fixed with 4% paraformaldehyde/4% sucrose (v/v) for 15 minutes at room temperature (RT). Next, cells were permeabilized with 0.2% triton in PBS for 10 minutes at RT. Unspecific binding sites were blocked by incubation in blocking buffer (PBS, 5% normal goat serum, 1% bovine serum albumin, 0.2% Triton) for 1 hour at RT. Primary antibodies were diluted in blocking buffer and incubated overnight at 4°C. Secondary antibodies, conjugated to Alexa-fluorochromes, were also diluted in blocking buffer and incubated for one hour at RT. Hoechst 33342 (Molecular Probes) was used to stain the nucleus before cells were mounted using DAKO (DAKO) fluorescent mounting medium and stored at 4°C. Cells were imaged at a 63x magnification using the Zeiss Axio Imager 2 equipped with Apotome. Used primary antibodies were: rabbit anti-GFAP (1:500; Abcam ab7260). Secondary antibodies that were used were: goat anti-guinea pig Alexa Fluor 647 (1:2000, Invitrogen A-21450); goat anti-rabbit Alexa Fluor 488 (1:2000, Invitrogen A- 11034); goat anti-rabbit Alexa Fluor 568 (1:2000, Invitrogen A-11036); goat anti-mouse Alexa Fluor 488 (1:2000, Invitrogen A-11029); goat anti-mouse Alexa Fluor 568 (1:2000, Invitrogen A-11031). Signals were quantified using FIJI software. The number of synaptic puncta was quantified per individual cell via manual counting and divided by the dendritic length of the neuron.

*Ehmt1*^+/+^ and *Ehmt1*^+/–^ littermates were harvested of both sexes at P50. Animals were deeply anesthetized with isoflurane, then decapitated and the brain was quickly extracted. The brains were fixed overnight in 4% PFA with 4% Sucrose in PBS at 4°C and kept in PBS at 4°C until slicing. The brains were embedded in 1.5% Agarose in PBS, after which 60 μm coronal sections were cut on a vibratome (Leica VT1000s). The slices were matched to the Allen Adult Mouse Brain Atlas (Atlas version 2, Oh et al., 2014; http://atlas.brain- map.org/atlas?atlas=1) and kept in PBS with 0.001% Sodium Azide at 4°C. Slices were permeabilized and blocked in 1% T-X PBS and 10% NGS for 2 hours at RT. Primary antibodies for S100B (287006, synaptic systems), and GFAP (ab7260, Abcam) were diluted in 0,4%T-X and 5% NGS and incubated overnight at 4 °C on a shaker. Slices were incubated with secondary antibodies Goat-anti-Chicken 568 (A11041 Invitrogen) and Goat-anti-rabbit 488 (A11034 Invitrogen) in 0,4%T-X and 5% NGS for 3 hours at RT. Hoechst 33342 (Molecular Probes) was used to stain the nucleus before cells were mounted using DAKO (DAKO) fluorescent mounting medium and stored at 4°C. Slices were imaged at 40x on a Zeiss AxioImager.Z1 with Apotome. A Zeiss EC Plan-Neofluar 5x / 0.16 M27 and Zeiss EC Plan-Neofluar 40x/0.75 M27 objectives were used and imaged with a Zeiss Axiocam 506. From each slice, we collected 2 non-overlapping 40x images of both layer 2/3 of the primary visual cortex and hippocampus region CA1. To compare intensity between all slides, all slices were imaged at the same intensity for all channels. Signals were quantified using in house script coded in FIJI software. Specifically, images were thresholded after which the total intensity of all pixels above threshold were measured (i.e., total S100B intensity in 568 and total GFAP intensity in the 488-channel displayed in Figure 2). For intracellular S100B intensity measures, the GFAP channel was used to generate a mask for each cell, which was used to measure the S100B intensity in the 568-channel.

### Quantification of mRNA by RT-PCR

RNA samples were isolated from both iPSCs or iNeurons using Nucleo Spin RNA isolation kit (Machery Nagel, 740955.250) according to the manufacturer’s instructions. RNA isolation from mouse prefrontal cortices were performed by Qiagen RNeasy Lipid Tissue Mini Kit (cat no 74804) after tissue homogenization via Qiazol (Qiagen, 79306) according to the protocol provided in the kit. RNA samples were converted into cDNAs by iScript cDNA synthesis kit (BIO-RAD, 1708891). cDNA products were cleaned up using the Nucleospin Gel and PCR clean-up kit (Machery Nagel, 740609.250). Human and mouse specific primers were designed by Primer3plus (http://www.bioinformatics.nl/cgi-bin/primer3plus/primer3plus.cgi) and IDT PrimerQuest (https://eu.idtdna.com) tools, respectively. Human and mouse primer sequences are given in Supplemental Table 9. qPCRs were performed in the 7500 Fast Real Time PCR System apparatus (Applied Biosystems) by using GoTaq qPCR master mix 2X with SYBR Green (Promega, A600A) according to the manufacturer’s protocol. The qPCR program was designed as following: after an initial denaturation step at 95°C for 10 min, PCR amplifications proceeded for 40 cycles of 95°C for 15 s and 60°C for 30 s and followed by melting curve. All samples were analyzed in duplicate in the same run and placed in adjacent wells. Reverse transcriptase-negative controls and no template-controls were included in all runs. The arithmetic mean of the Ct values of the technical replicas was used for calculations. Relative mRNA expression levels were calculated using the 2-ΔΔCt method with standardization to housekeeping gene.

### RNA-seq

RNA samples were isolated from human astrocyte culture when they are 4 weeks old starting from culturing in the astrocyte medium. RNA from three biological replicates of control and *EHMT1*^+/–^ human astrocytes were isolated using the NucleoSpin RNA isolation kit (Machery Nagel, 740955.250) according to the manufacturer’s instructions. RNA yield was quantified with a NanoDrop Spectrophotometer (NanoDrop Technologies, Wilmington, DE, USA). All samples had an RNA Integrity Number (RIN) > 9. Strand-specific cDNA libraries were prepared using the NEBNext Ultra II Directional RNA Library Prep Kit, with strandedness reversed during insertion of dIDT UMI adapters. Libraries were sequenced on an Illumina NovaSeq 6000 (GenomeScan B.V., Leiden), generating approximately 40 million paired-end reads per sample. In the resulting libraries, Read 1 (R1) corresponded to the sense strand, and Read 3 (R3) to the antisense strand. The unique molecular identifier (UMI) located in Read 2 (R2) was transferred to the RX tag in the BAM file for UMI-aware deduplication. Adapter and quality trimming were performed using fastp, and reads were aligned to the GRCh38 reference genome using HISAT2 with the --rna-strandness FR option. The resulting BAM files were sorted and indexed with SAMtools, followed by UMI-based deduplication using UMI-tools. Gene-level quantification was performed with featureCounts (Subread package).

In addition, co-cultures of wild-type rat astrocytes with iPSC-derived neurons, either carrying the *EHMT1*^+^/^–^ mutation or their isogenic controls^15^, were included in the RNA-seq analysis, with three technical replicates per condition. Total RNA was isolated using the Zymo Quick-RNA Microprep Kit, and RNA integrity was assessed with Agilent’s High Sensitivity TapeStation (RIN 7.5–9.6). cDNA libraries were prepared using the NEBNext Ultra II RNA Library Prep Kit for Illumina with poly(A) mRNA enrichment, and sequenced on an Illumina NovaSeq 6000 (GenomeScan B.V., Leiden), producing approximately 40 million 150 bp paired-end reads per sample. Raw reads were processed following the same pipeline described above, except that Seal (BBTools) was first used to separate rat and human reads by alignment to their respective reference genomes (Rnor_6.0 and GRCh38) using the ambig=first setting. Species-specific reads were then aligned with HISAT2 using the --rna-strandness RF option, followed by sorting, indexing, UMI-based deduplication (UMI-tools), and gene-level quantification (featureCounts).

### Differential expression analysis

The gene-level counts obtained from featureCounts were used as input for differential expression analysis, GO enrichment, and targeted gene set evaluation. Lowly expressed genes with fewer than 10 reads in fewer than two samples were excluded. Normalization and differential expression analysis were carried out using DESeq2, and genes with log2 fold change > 1 or log2 fold change < -1 and Benjamini-Hochberg adjusted p-value < 0.05 were considered significantly differentially expressed. Gene Ontology enrichment analysis was performed for Biological Processes, Molecular Functions, and Cellular Components categories using topGO with Fisher’s exact test and adjusted for multiple testing using the Benjamini-Hochberg method. GO terms annotated to fewer than 10 or more than 500 genes were excluded to retain informative gene sets while excluding terms that are too small or too broad for meaningful interpretation. Visualization and hierarchical clustering were performed using the ComplexHeatmap R package. RNAseq data are available on https://doi.org/10.34973/tb65-2n91

### Micro-electrode array recordings and data analysis

All recordings were performed using the 24-well MEA system (Multichannel Systems, MCS GmbH, Reutlingen, Germany) as described before (16). Spontaneous electrophysiological activity of neuronal networks was recorded at 37°C and constant flow of humidified gas (5% CO_2_ and 95% O_2_). The raw signal was sampled at 10 kHz and filtered with a high-pass filter (i.e., 2nd order Butterworth, 100 Hz cut-off frequency) and a low- pass filter (i.e., 4th order Butterworth, 3500 Hz cut-off frequency). The threshold for detecting spikes was set at ± 4.5 standard deviations. We performed off-line data analysis by using Multiwell Analyzer (i.e., software from the 24-well MEA system that allows the extraction of the spike trains) and a custom software package named SPYCODE developed in MATLAB (The Mathworks, Natick, MA, USA), that allows the extraction of parameters describing the network activity. The parameters extracted using Multiwell analyser include the mean firing rate (MFR), which is the average total number of spikes detected per electrode over time (spikes/second in Hz). The MFR is averaged per well for all electrodes. We detected bursts based on the maximum inter-spike interval (ISI) to start or end a burst (i.e., for the whole spike train, with a maximum ISI of 50 ms). Spikes were included in the burst if the ISI is shorter than 50 ms, whereas the burst was ended if the ISI is larger than 50 ms. Bursts that were less than 100 ms apart were merged. All bursts that had a duration of less than 50 ms or had less than 4 spikes were removed from the detection. Network bursts were detected when a burst occurs in more than 80% of the active channels and presented as network burst/minute and the duration in seconds. Not active wells (i.e., MFR < 0.16 Hz in at least 3 channels to be called active), wells that had no network burst at DIV 28, wells where network bursts were detected in less than 50% of the channels and wells where the firing rate decreased over development were rigorously discarded.

### Single cell electrophysiology

Single cell electrophysiology was performed at DIV28 as described previously (16). Cells were recorded in a recording chamber on the stage of an Olympus BX51WI upright microscope (Olympus Life Science, PA, USA) equipped with infrared differential interference contrast optics, an Olympus LUMPlanFL N 40x water- immersion objective (Olympus Life Science, PA, USA) and kappa MXC 200 camera system (Kappa optronics GmbH, Gleichen, Germany) for visualization. Recordings were made using a SEC-05X amplifier (NPI Electronic GmbH, Tamm, Germany), low-pass filtered at 3 Khz and digitized at 10kHz using a 1401 acquisition board and recorded with Signal (CED, Cambridge, UK). Recordings were not corrected for liquid junction potential (± 10 mV). A continuous flow of oxygenated (95% O2/5% CO2) artificial cerebrospinal fluid (ACSF) containing (in mM) 124 NaCl, 1.25 NaH2PO4, 3 KCl, 26 NaHCO3, 11 Glucose, 2 CaCl2, 1 MgCl2 (adjusted to pH 7.4), warmed to 30°C, flowed through the recording chamber. Patch pipettes (6-8 MΩ) were pulled from borosilicate glass with filament and fire-polished ends (Science Products GmbH, Hofheim, Germany) using the PMP-102 micropipette puller (MicroData Instrument, NJ, USA). Pipettes were filled with a potassium-based solution containing (in mM) 130 K-Gluconate, 5 KCl, 10 HEPES, 2.5 MgCl2, 4 Na2-ATP, 0.4 Na3-ATP, 10 Na- phosphocreatine, 0.6 EGTA (adjusted to pH 7.25 and osmolarity 290 mosmol) for recordings of intrinsic electrophysiological properties in current clamp mode, where the resting membrane potential (V_rmp_) was determined after achieving whole-cell configuration. Cells were selected on a V_rmp_ of -55 mV and lower. All further recordings were performed at a holding potential of -60 mV. Passive membrane properties were determined via a 0.5 s hyperpolarizing current of -25 pA. Action potential (AP) characteristics were determined from the first AP elicited by a 0.5 s depolarizing current injection just sufficient to reach AP threshold. Spontaneous action potential-evoked postsynaptic currents (sEPSC) were recorded in ACSF without additional drugs at a holding potential of −60 mV. Intrinsic electrophysiological properties were analysed using Signal and MatLab (MathWorks, MA, USA), while spontaneous EPSCs were analysed using MiniAnalysis (Synaptosoft Inc, Decatur, GA, USA).

### Oxidative stress assay

Control and EHMT1-deficient astrocytes were plated on glass coverslips in a 24-well plate coated with 0.0125% PEI (Sigma- Aldrich) and were cultured in high-glucose astrocyte media (high-glucose DMEM (Gibco, cat. no. 11965-092) with 15% (v/v) FBS (Sigma-Aldrich, cat. no. F2442) and 1% (v/v) penicillin/streptomycin (Sigma-Aldrich, cat. no. P4333)) that was refreshed every 2-3 days. At approximately 80% confluence, the CellROX® assay (Invitrogen cat. no. C10444) was performed according to the manufacturer’s protocol. Rotenone, a complex I inhibitor, was used as a positive control to induce oxidative stress in control astrocytes 30 minutes and 6 hours using a concentration of 100 nM. Thirty minutes before fixation CellROX® green was added to the media at a final concentration of 5 μM, and cells were incubated at 37°C/5% CO2. Cells were washed three times with ice-cold PBS and then fixated with 4% PFA in PBS for 15 minutes. Hoechst 33342 (Molecular Probes) was used to stain the nuclei before cells were mounted using DAKO (DAKO) fluorescent mounting medium. We imaged at a 63x magnification using the Zeiss Axio Imager Z1 equipped with Apotome. All conditions within a batch were acquired with the same settings to compare signal intensities between different experimental conditions. Fluorescent signals were quantified using FIJI software. The intensity of the CellROX ® green dye was calculated by: integrated density – (Area of selected cell X Mean fluorescence of background readings).

### Statistics

The statistical analysis for all experiments was performed using GraphPad Prism 8 (GraphPad Software, Inc., CA, USA). *P* values less than 0.05 for the different experimental conditions were considered significant. Statistical analysis of electrophysiological data was performed with 1-way ANOVA and post hoc Bonferroni (normal distribution) or Kruskal-Wallis ANOVA with post hoc Dunn’s (not normally distributed) correction for multiple testing. When comparing means of 2 different groups, we determined significance by means of a paired *t* test (paired data) or unpaired *t* test (unpaired data, 2-tailed). Data are presented as Mean ± SEM.

### Study approval

This study was approved by the regional medical ethical committees of Radboud University Medical Center and Boston Children’s Hospital, under NL65650.091.18 and IRB-P00032696, respectively. Informed consent was obtained by legal representatives, since most individuals are incapacitated because of their intellectual disability. Experiments involving patient-derived hiPSCs were conducted with informed consent and approval from the Medical Ethical Committee of Radboud University Medical Center, Nijmegen (2018–4525).

### Data availability

All data are available on reasonable request. RNAseq data are available on https://doi.org/10.34973/tb65-2n91 Values for all data points in graphs are reported in the Supporting Data Values file.

## Author contributions

TK and NNK conceptualized and supervised the study. KVK, SW and JK wrote the original draft of this manuscript and were responsible for the visualization of the data. NNK, DS and SW were responsible for all preclinical work. TK, KVK and JK were repsonsible for the clinical work. NNK and TK reviewed and edited the manuscript. KVK, SW, JK, BM, CGJ, ZF, BR, AOL, EH, MF, KL, MN, BL, AO, MPP, JJ, LVD, IVDW, CS, WO designed and performed experiments; KW perfomed RNA -seq data analysis. NNK, TK, SS, FMSDV, SAK, JIME, HVB, DS supervised experiments and gave feedback on manuscript. Order of shared first authorship was decided after agreement between all first authors and last authors TK and NNK.

## Conflict of interest

The authors have declared that no conflict of interest exists.

## Funding support

- ERA-NET NEURON-102 SYNSCHIZ grant (NWO) 013-17-003 4538 (to DS).
- The Netherlands Organization for Health Research and Development ZonMw VIDI 91718310 and ZonMw-Open 09120012110034 (to TK).
- BRAINmodel ZonMW PSIDER program 10250022110003 (to NNK).
- Dutch Research Council grant 015.014.036 (to TK).
- SFARI grant 890042 (to NNK).
- China scholarship council 201806210076 (to SW).
- The Netherlands Organ-on-Chip Initiative, an NWO Gravitation project funded by the Ministry of Education, Culture and Science of the government of the Netherlands (024.003.001) to FDV and SAK.

## Supplementary Material

Supplemental data

Unedited blot and gel images

Supplemental table 5

Supplemental table 7

Supplemental table 8

Supporting data values

## Version 1 06/30/2026

In-Press Preview

## Version 2 08/17/2026

Electronic publication
