# Supplementary material for: Astrocytes contribute to olanzapine-mediated reversal of kleefstra syndrome-associated neurodevelopmental regression
Source: J Clin Invest. 2026 Jun 30;136(16):e195803. doi: 10.1172/JCI195803 (PMC13476134; doi:10.1172/JCI195803)
Supplement: Supplementary file 1 [file jci-136-195803-s070.pdf]

Astrocytes contribute to Olanzapine-mediated reversal of Kleefstra Syndrome-Associated Neurodevelopmental Regression

Karlijn Vermeulen-Kalk[1,2,3]*, Shan Wang[4]*, Joost Kummeling[1,4]*, Britt Mossink[1,4], Kim Wijnant[1], Carlos Gonzales Jiménez[1], Zoe Frazier[5], Brian Rozumny[6], Anne O'Donnell-Luria[7,8], Ellen Hanson[6,9], Monica Frega[1,4], Katrin Linda[1,4], Moritz Negwer[1,4], Bas Lendemeijer[11], Astrid Oudakker[1], Monica Pop-purceleanu[10], Joost Janzing[10], Linde van Dongen[1,4], Femke M.S. de Vrij[11,12], Steven A. Kushner[11,13], Ilse van der Werf[4,15], Chantal Schoenmaker[1], Wouter Oomens[2], Siddharth Srivastava[5,9], Jos Egger[2,4,14], Hans van Bokhoven[1,4], Dirk Schubert[4,15], Nael Nadif Kasri[1,4#], Tjitske Kleefstra[1,2,16#].

[1]Department of Human Genetics, Radboud UMC, Nijmegen, the Netherlands
[2]Centre of Excellence for Neuropsychiatry, Vincent van Gogh Institute for Psychiatry, Venray, The Netherlands
[3]Department for Intellectual Disabilities, Karakter Child and Adolescent Psychiatry, Ede, the Netherlands
[4]Donders Institute for Brain, Cognition and Behaviour, Nijmegen, the Netherlands
[5]Rosamund Stone Zander Translational Neuroscience Center, Department of Neurology, Boston Children's Hospital, Boston, MA 02115, USA
[6]Division of Developmental Medicine, Boston Children's Hospital, Boston, MA 02115, USA
[7]Division of Genetics and Genomics, Boston Children's Hospital, Boston, MA 02115, USA
[8]Program in Medical and Population Genetics, Broad Institute of MIT and Harvard, Cambridge, Massachusetts 02142, USA
[9]Department of Neurology, Boston Children's Hospital, Boston, MA 02115, USA
[10]Department of Psychiatry, Radboud University Medical Center, Nijmegen, the Netherlands
[11]Department of Psychiatry, Erasmus MC University Medical Center, Rotterdam, the Netherlands
[12]ENCORE Expertise Center for Neurodevelopmental Disorders, Erasmus MC, Rotterdam, the Netherlands
[13]Department of Psychiatry, Columbia University, New York, NY 10032
[14]Stevig Specialized and Forensic Care for People with Intellectual Disabilities, Dichterbij, Oostrum, the Netherlands
[15]Department of Medical Neurosciences, Radboud UMC, Nijmegen, the Netherlands
[16]Department of Clinical Genetics, Erasmus MC University Medical Center, Rotterdam, the Netherlands

Supplementary figures: S1-11

Supplementary tables: 1-11

Supplemental Materials and Methods

Supplemental figures

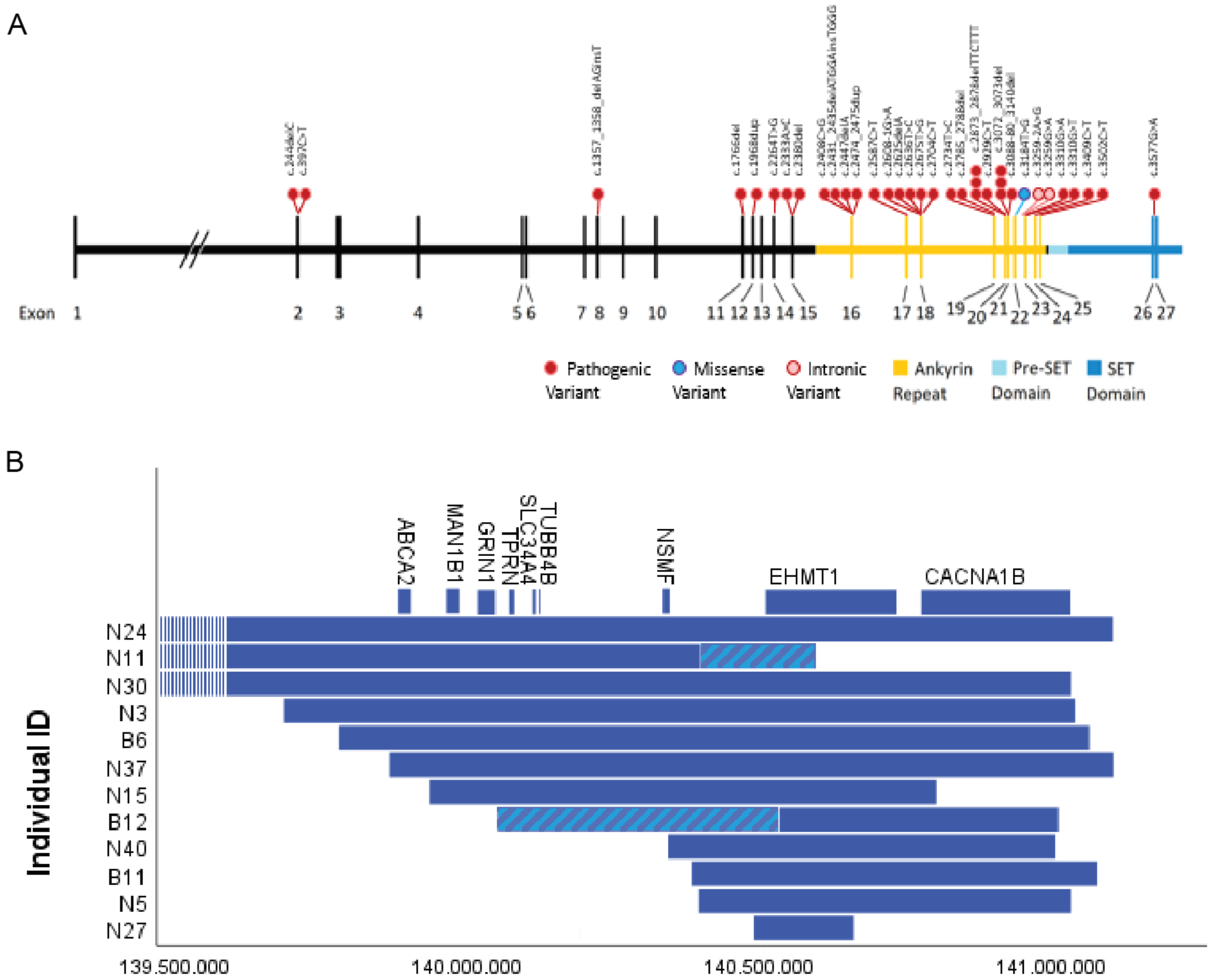

Figure S1. Overview of *EHMT1* variants in KLEFS1 individuals included in this study. **(A)** Thirty-eight Individuals have a pathogenic *EHMT1* variant. Two comprise intronic variants (c.3259-2A>G and c.3259G>A), one a missense variant (c.3184T>G), the remaining truncating variants. Variant c.2873_2878delTTCTTT was present in two siblings, c.3072_3073del recurrent in two non-related individuals. **(B)** Schematic overview of 9q34.3 deletions (genome assembly GRCh37)**,** ranging from largest (3,2Mb) to smallest (165kb) (partially) covering *EHMT1* and flanking genes in KLEFS1 individuals included in this study. Anterior deletion breakpoint N24, N11, N30, not exactly known; dashed bar in N11 and B12 represent a range of uncertain size. N39 has an intragenic deletion of exon 5-17 (not shown). Locations of the deletions of N04, N20, and N25 not shown in detail due to incomplete data.

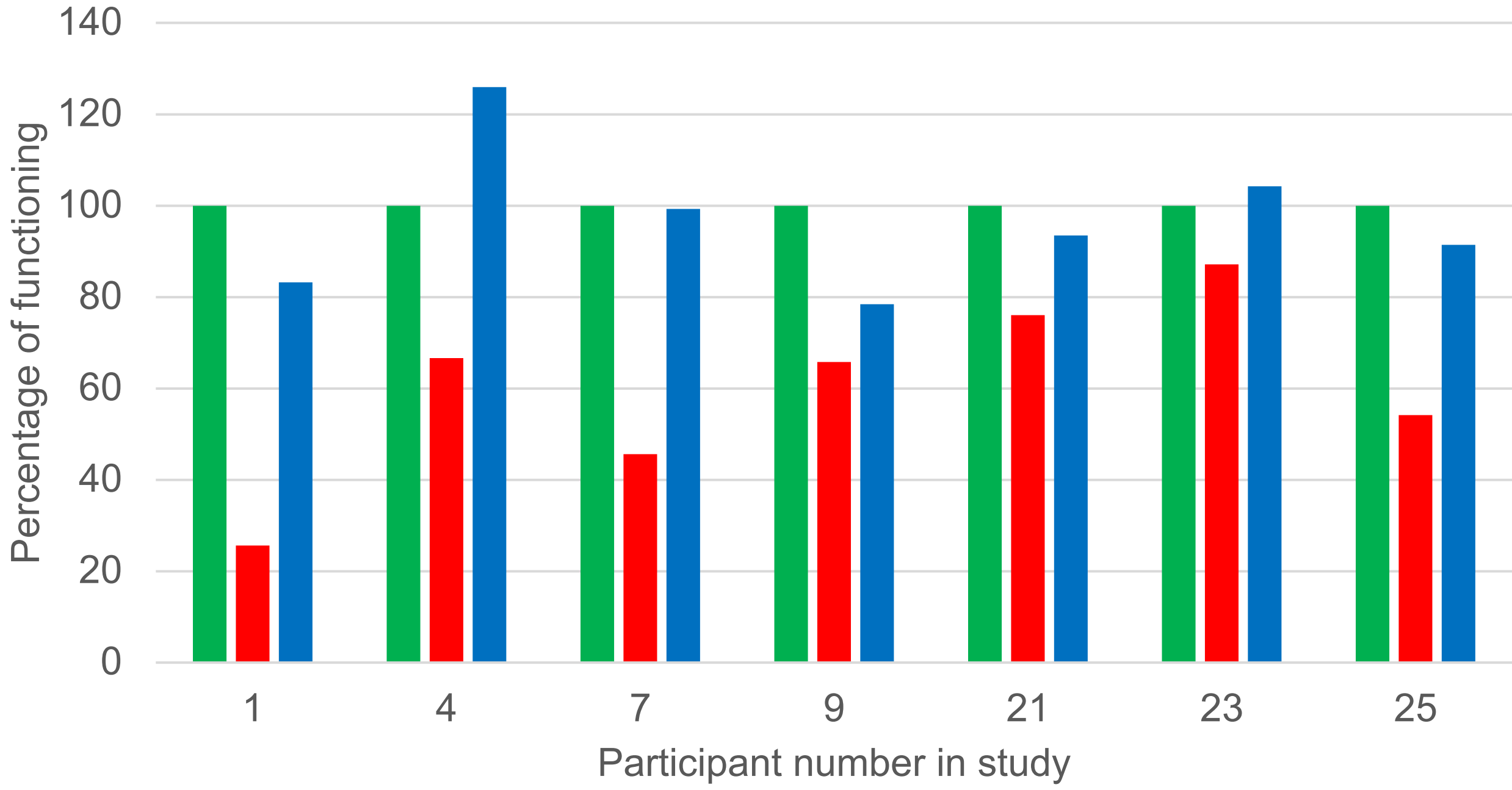

Figure S2. Decline and recovery plotted as a percentage of initial functioning for all participant for which records were available before and after regression.

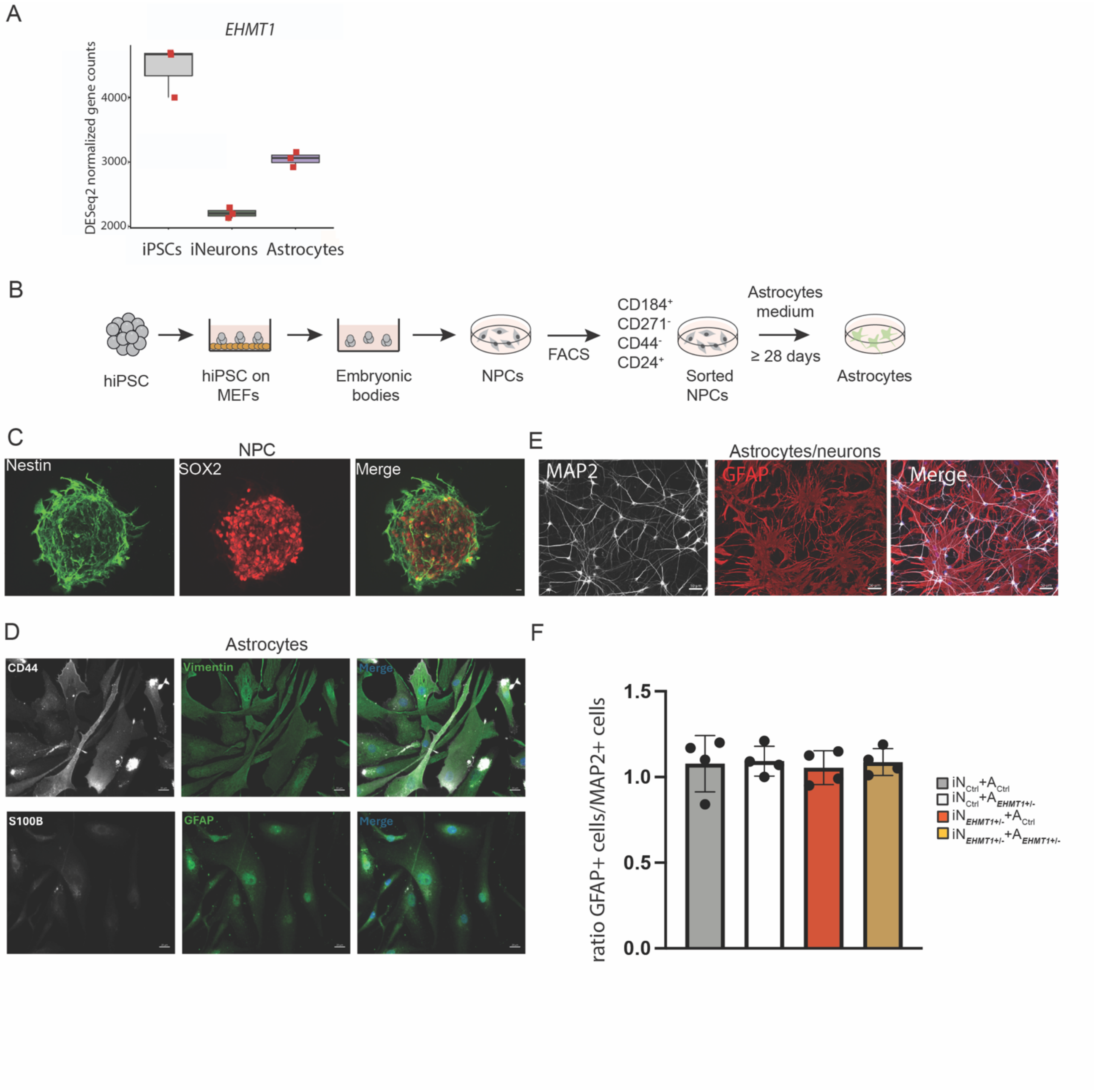

Figure S3. Characterization of *EHMT1*$^{+/-}$ astrocytes

(A) Expression profile *EHMT1* in iPSCs, iNeurons and iPSC-induced astrocytes extracted from transcriptomic data in Wijnant K. et al.[13] **(B)** Differentiation pipeline of iPSC-derived astrocytes. **(C)** Representative images of neuron progenitor cells (NPCs) showing positive signals for Nestin (green) and SOX2 (red). Scale bar = 10 µm. **(D)** Representative images of astrocytes showing positive signals for CD44, Vimentin, GFAP and S100β (green). **(E)** Representative images of astrocytes(GFAP)-neuron(MAP2) co-cultures. Scale bar = 50 µm. **(F)** Quantification of GFAP positive (astrocytes) and MAP2 positive (neurons) cells in the indicated networks, n=4.

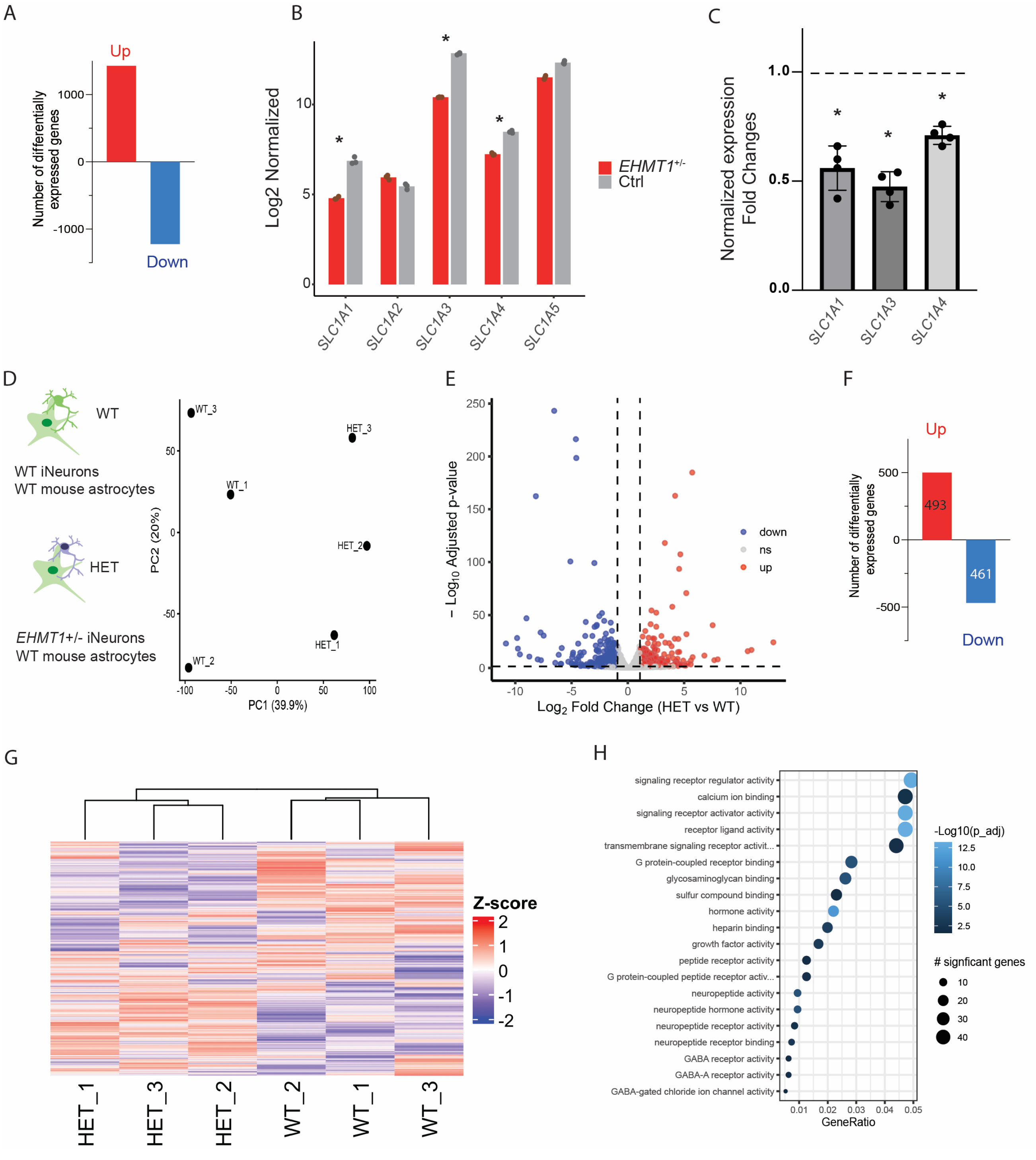

Figure S4. Analysis of *EHMT1*$^{+/-}$ astrocytes and neurons transcriptome. (A) Amount of differentially expressed genes up- and downregulated in *EHMT1*$^{+/-}$ astrocytes. **(B)** normalzied expression of key glutamate transporters genes obtaind from RNA-seq data set. **(C)** qPCR quantification of glutamate transporters genes *SLCA1*, *SLCA3* and *SLCA4* in ctr and *EHMT1*$^{+/-}$ astrocytes. Gene expression normalized to control values, n=4. **(D)** Principal component analysis (PCA) of RNA-Sequencing data from control (Ctrl) and *EHMT1*$^{+/-}$ iNeurons cultured on mouse control astrocytes, n=3 independent cultures. **(E)** Volcano plots showing differentially expressed genes (DEGs) between *EHMT1*$^{+/-}$ and control iNeurons. Relative to the control, significantly up- or down-regulated genes are shown above the black dashed line in red and blue (FDR < 0.05; log2(FC) > 1; log2(FC) < -1). **(F)** Amount of differentially expressed genes up- and downregulated in *EHMT1*$^{+/-}$ iNeurons. **(G)** Heatmap showing relative normalized gene expression across samples. Normalized

counts were log_2-transformed and scaled per gene to Z-scores, representing relative expression levels. Both genes and samples were hierarchically clustered based on Z-scores. **(H)** GO term analysis of molecular function. Data represent means ± SEM. *P < 0.05. Unpaired Student's t-test (B and C).

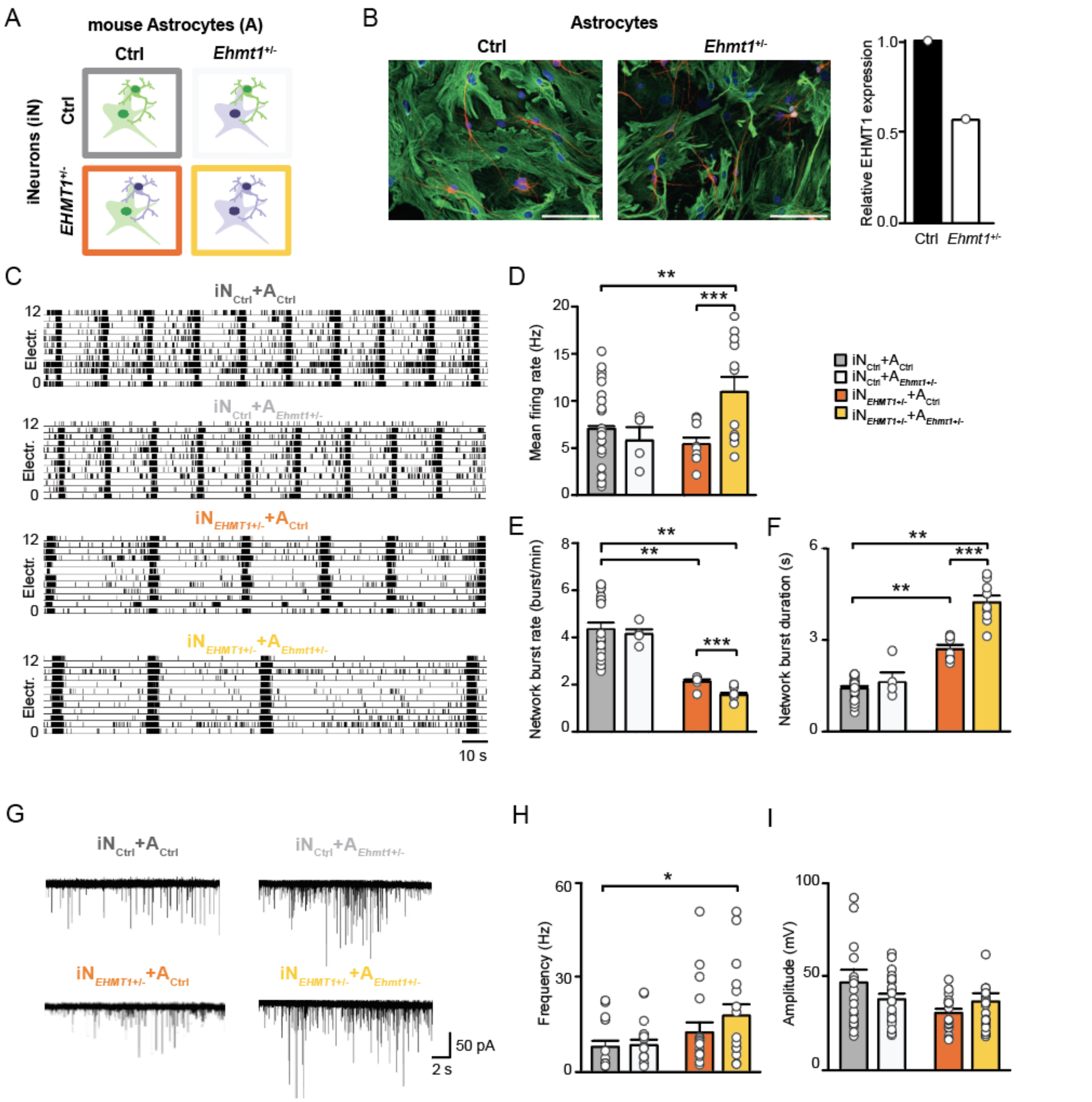

Figure S5. Mouse *Ehmt1*$^{+/-}$ astrocytes induce neuronal network hyperactivity in *EHMT1*$^{+/-}$ neurons

(A) Neurons derived from control or *Ehmt1*$^{+/-}$ iPSCs are co-cultured together with either control (Ctrl) or mouse *Ehmt1*$^{+/-}$ astrocytes. These four composite networks (i.e. iN_{Ctrl} + A_{Ctrl}, iN_{Ctrl} + $A_{Ehmt1+/-}$, $iN_{EHMT1+/-}$ + A_{Ctrl} and $iN_{EHMT1+/-}$ + $A_{Ehmt1+/-}$) are shown in grey, light grey, orange and yellow respectively. **(B)** Representative figures of iN_{Ctrl} + A_{Ctrl} and $iN_{EHMT1+/-}$ + $A_{Ehmt1+/-}$ networks stained for MAP2 (red) and GFAP (green) at DIV 21 (scale bar 100 µm). EHMT1 expression, quantified with immunocytochemisry, is significantly reduced in *Ehmt1*$^{+/-}$ mouse astrocytes. **(C)** Representative raster plots showing 3 min of recording of the spontaneous activity exhibited in composite networks on MEA at DIV 28. (**D-F**) Graphs respectively showing **(D)** mean firing rate, **(E)** network burst rate and **(F)** network burst duration of composite networks. Two-way ANOVA with post hoc Bonferroni correction or Kruskal Wallis ANOVA with Dunn's correction for multiple testing was performed between composite networks (number of MEAs recorded from two independent neuronal preparations (n): iN_{Ctrl} + A_{Ctrl} n = 22, iN_{Ctrl} + $A_{Ehmt1+/-}$ n = 9, $iN_{EHMT1+/-}$ + A_{Ctrl} n = 7 and $iN_{EHMT1+/-}$ + $A_{Ehmt1+/-}$ n=9). **(G-I)** Data collected from single-cell patch clamp. **(G)** Representative example traces (i.e. 20 s) of spontaneous excitatory postsynaptic currents (sEPSCs) received by composite networks at DIV 28. **(H-I)** Graphs respectively showing **(H)**

sEPSC frequency and **(I)** sEPSC amplitude. Data represent means ± SEM. Two-way ANOVA with post hoc Bonferroni correction or Kruskal Wallis ANOVA with Dunn's correction for multiple testing was performed between composite networks (number of cells recorded from 3 independent neuronal preparations (n): iN_{Ctrl} + A_{Ctrl} n = 17, iN_{Ctrl} + $A_{\mathit{Ehmt1+/}}$ n = 13, $iN_{\mathit{EHMT1+/-}}$ + A_{Ctrl} n = 26 and $iN_{\mathit{EHMT1+/-}}$ + $A_{\mathit{Ehmt1+/-}}$ n = 26). Data represent means ± SEM. * P<0.05, ** P<0.01, *** P<0.001. DIV: Days in vitro.

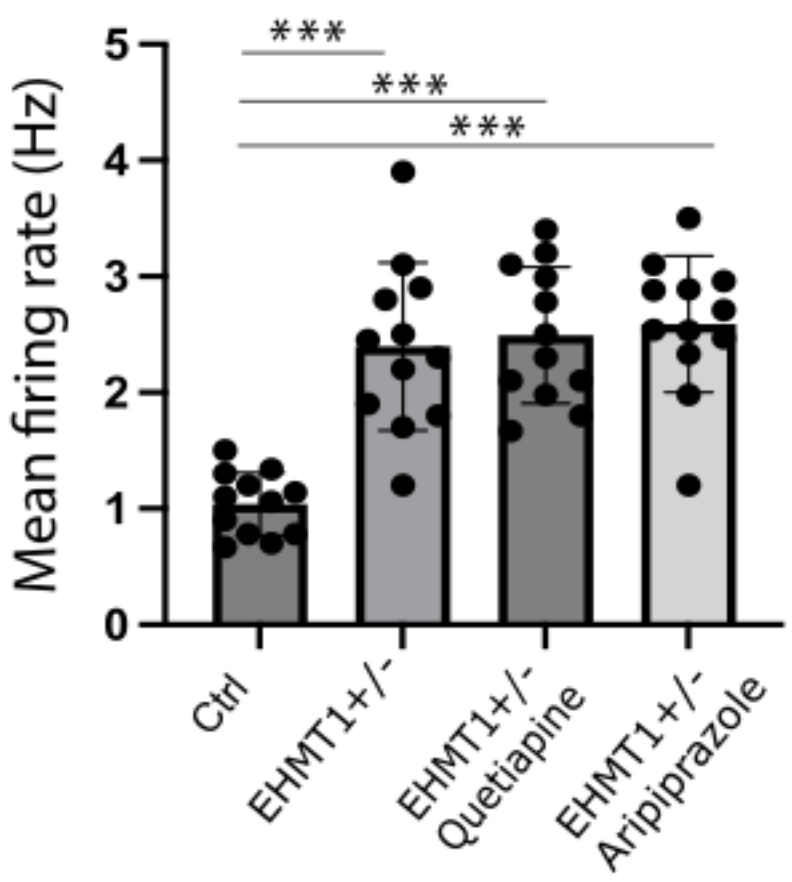

Figure S6. Quantification of mean firing rate exhibited by *EHMT1*$^{+/-}$ astrocyte-neuron co-cultures on MEAs treated with 10 µM quetiapine or aripiprazole for one week. n = 12, 3 independent experimenst. Data represent means ± SEM. ***P < 0.0001, one-way ANOVA.

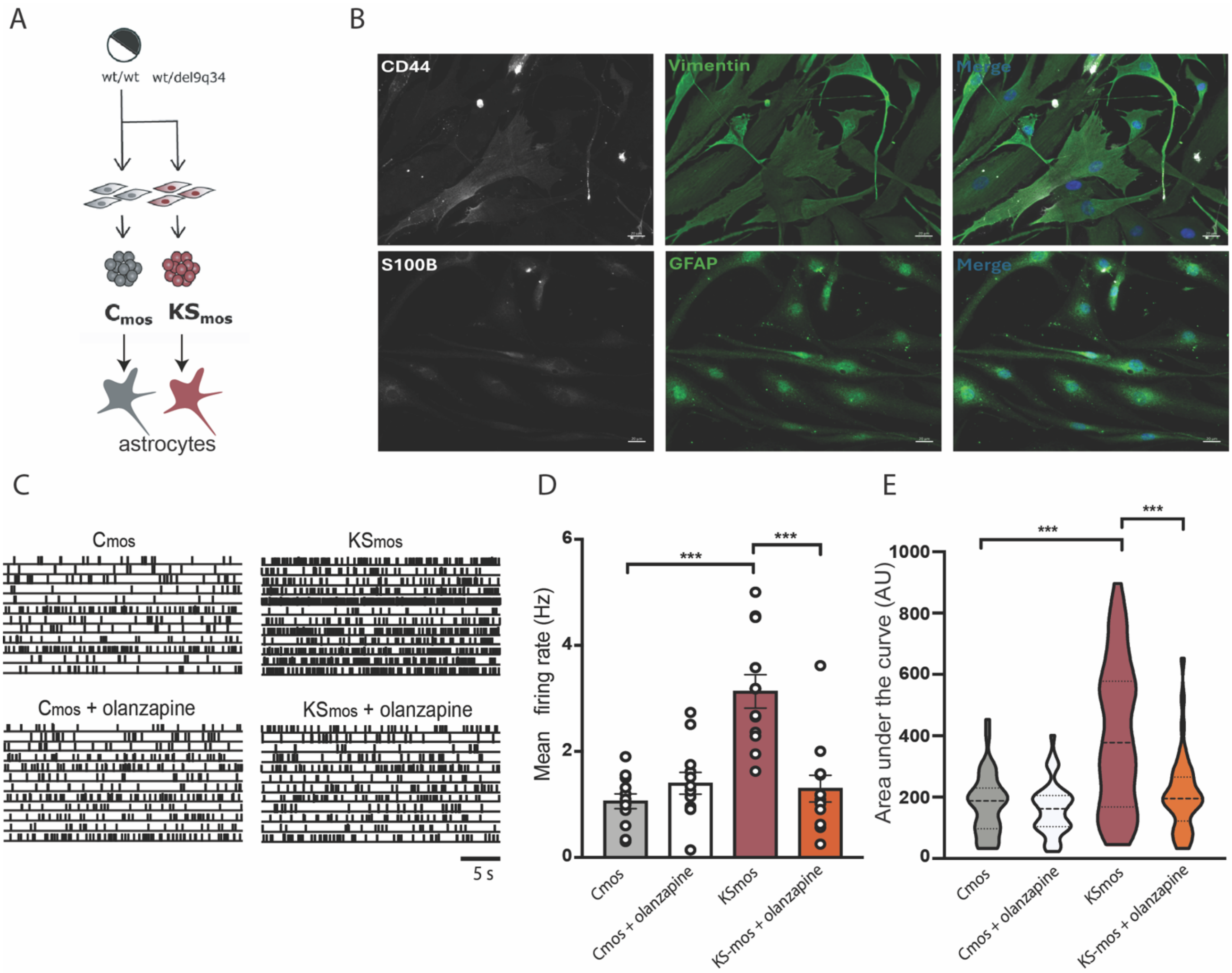

Figure S7. (A) Patient iPS cells used in this study, described in *Frega* et al[14]. **(B)** Representative images of astrocytes derived from patuent line (KSmos) showing positive signals for CD44, Vimentin, GFAP and S100β (green). **(C)** Representative raster plot and **(D)** quantification of mean firing rate exhibited by control or *EHMT1*$^{+/-}$ neuron-astrocytes co-cultures with and without olanzapine at DIV21, n = 12. Data represent means ± SEM. *P < 0.05, **P < 0.01, ***P < 0.001. One-way ANOVA test and post hoc Bonferroni correction. **(E)** Quantification of calcium wave from control (Cmos) and EHMT1$^{+/-}$ (Ksmos) astrocytes treated with or without olanzapine upon 50 μM glutamate stimulation, n = 40-60 cells from 4 independent experiments. Data represent means ± SEM. *P < 0.05, **P < 0.01, ***P < 0.001. One-way ANOVA test and post hoc Bonferroni correction.

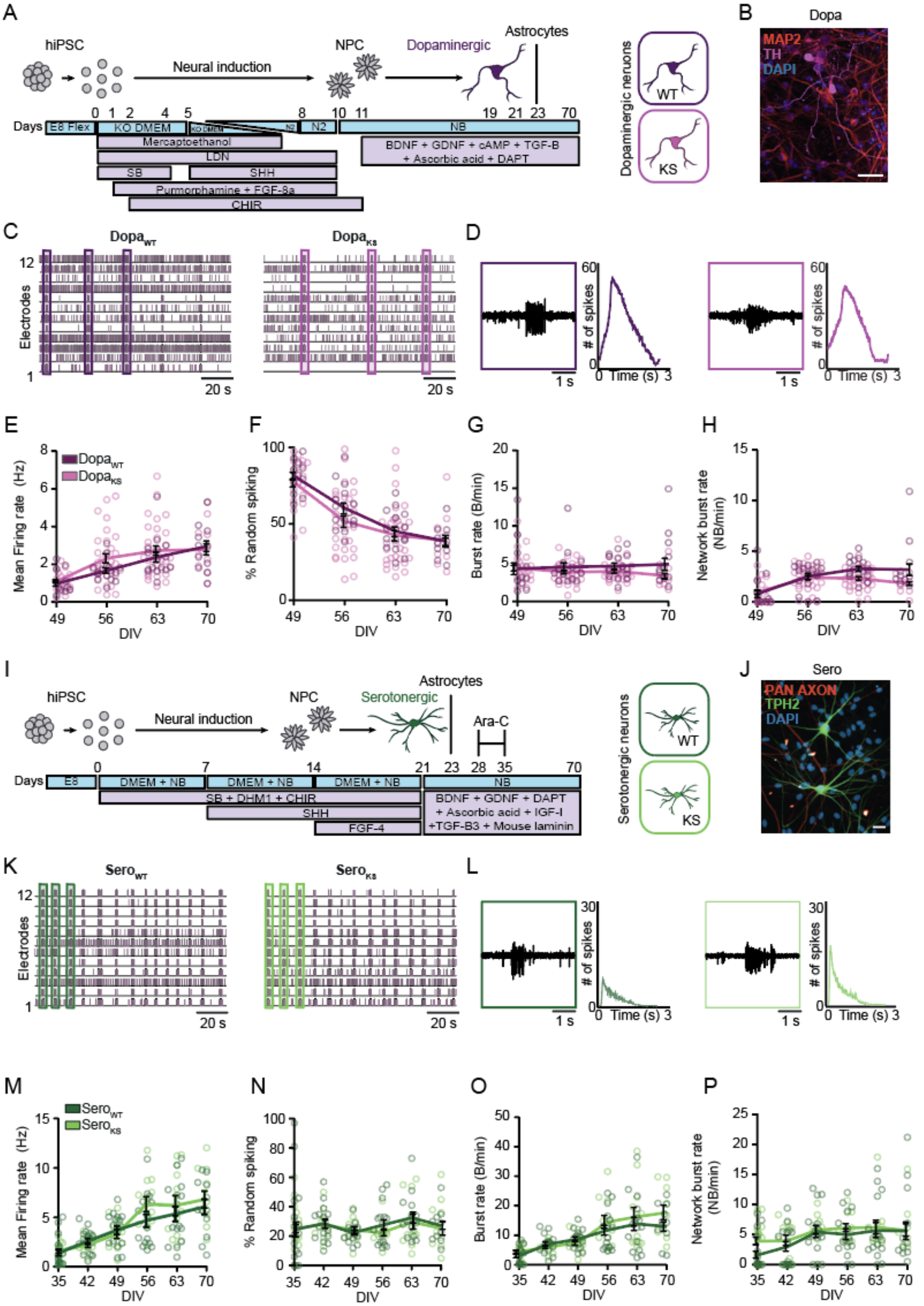

Figure S8. Dopaminergic and serotonergic neuronal network activity is not affected by *EHMT1* haploinsufficiency. (A) The differentiation protocol for the generation of dopaminergic neurons and **(B)** immunostaining-based characterisation with neuronal marker MAP2 co-labelled with dopaminergic marker TH at DIV 71. **(C)** Representative raster plots showing 120 seconds of

electrophysiological activity recorded from 12 electrodes of dopaminergic networks at DIV 70. **(D)** Representative burst shape of one electrode, followed by the average network burst shape of all electrodes in that respective well. (E-H) Quantification of neuronal activity including (mean firing rate, percentage random spiking, burst rate and network burst rate). **(I)** The differentiation protocol for the generation of serotonergic neurons and **(J)** immunostaining-based characterisation with TPH2 at DIV 71. **(K)** Representative raster plots showing 120 seconds of electrophysiological activity recorded from 12 electrodes of serotonergic networks at DIV 70. **(L)** Representative burst shape of one electrode, followed by the average network burst shape of all electrodes in that respective well. **(M-P)** Quantification of neuronal activity including (mean firing rate, percentage random spiking, burst rate and network burst rate). $Dopa_{WT}$: n = 16; $Dopa_{KS}$: n = 16; $Sero_{WT}$: n = 16; and $Sero_{KS}$: n = 16 recordings from individual MEA wells from 3 independent neuronal preparation). Mixed effect model was performed between WT and *EHMT1*-deficient networks over time; p-values were corrected for multiple comparisons using Bonferroni's. All data represents means ± SEM. DIV: Days in vitro. * $p < 0.05$; ** $p < 0.01$; *** $p < 0.001$.

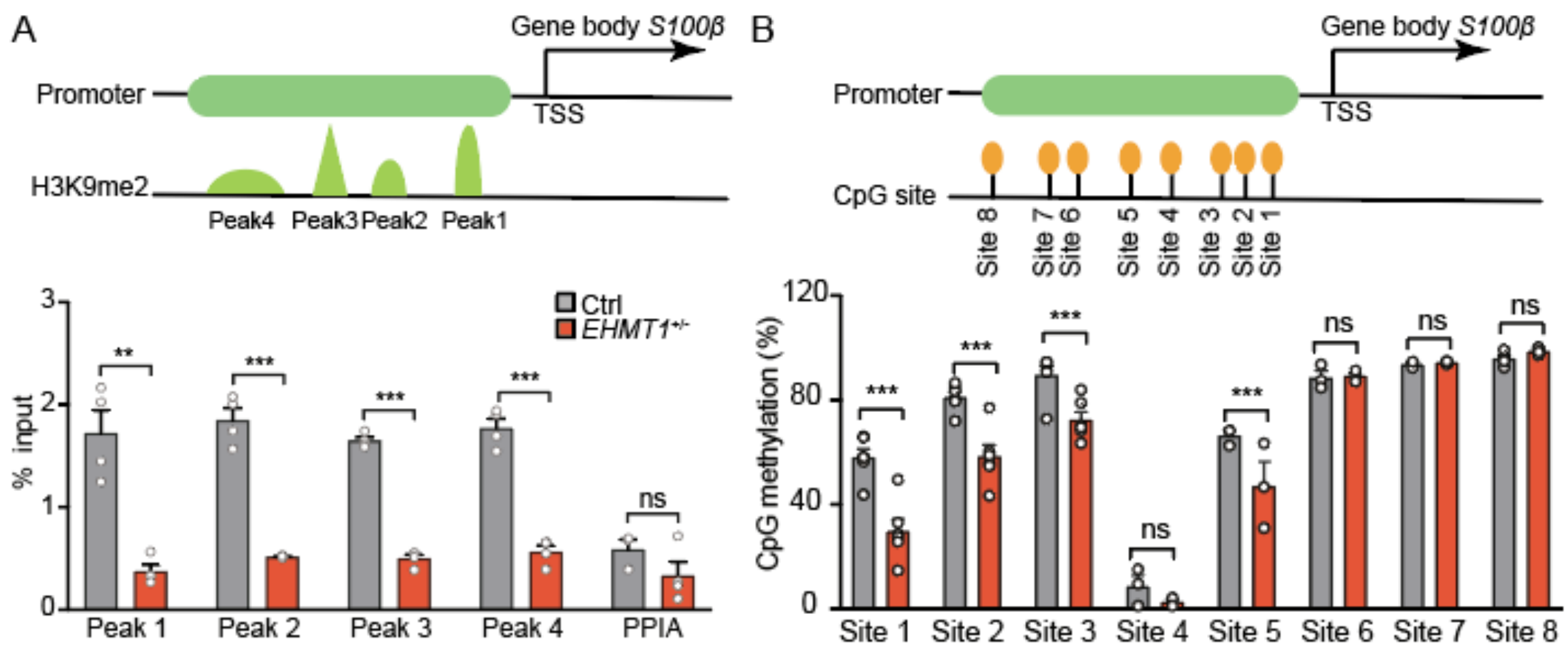

Figure S9. Reduced H3K9me2 and DNA methylation at promotor *S100β* in *EHMT1*$^{+/-}$ human astrocytes (A) Scheme showing 4 potential methylation regions of H3K9me2 in the promoter of *S100β* (upper panel). CUT & RUN assay of H3K9me2 followed by promoter-specific (*S100β*, PPIA) qPCR performed in control and *EHMT1*$^{+/-}$ astrocytes (n = 4). (lower panel) **(B)** Scheme showing 8 potential DNA methylation sites in the promoter of *S100β* (upper panel). Bisulfite-conversion assay followed by sanger sequencing (lower panel) n = 3-6. Data represent means ± SEM. *P < 0.05, **P < 0.01, ***P < 0.001. Multiple T-test with *post-hoc* Bonferroni correction.

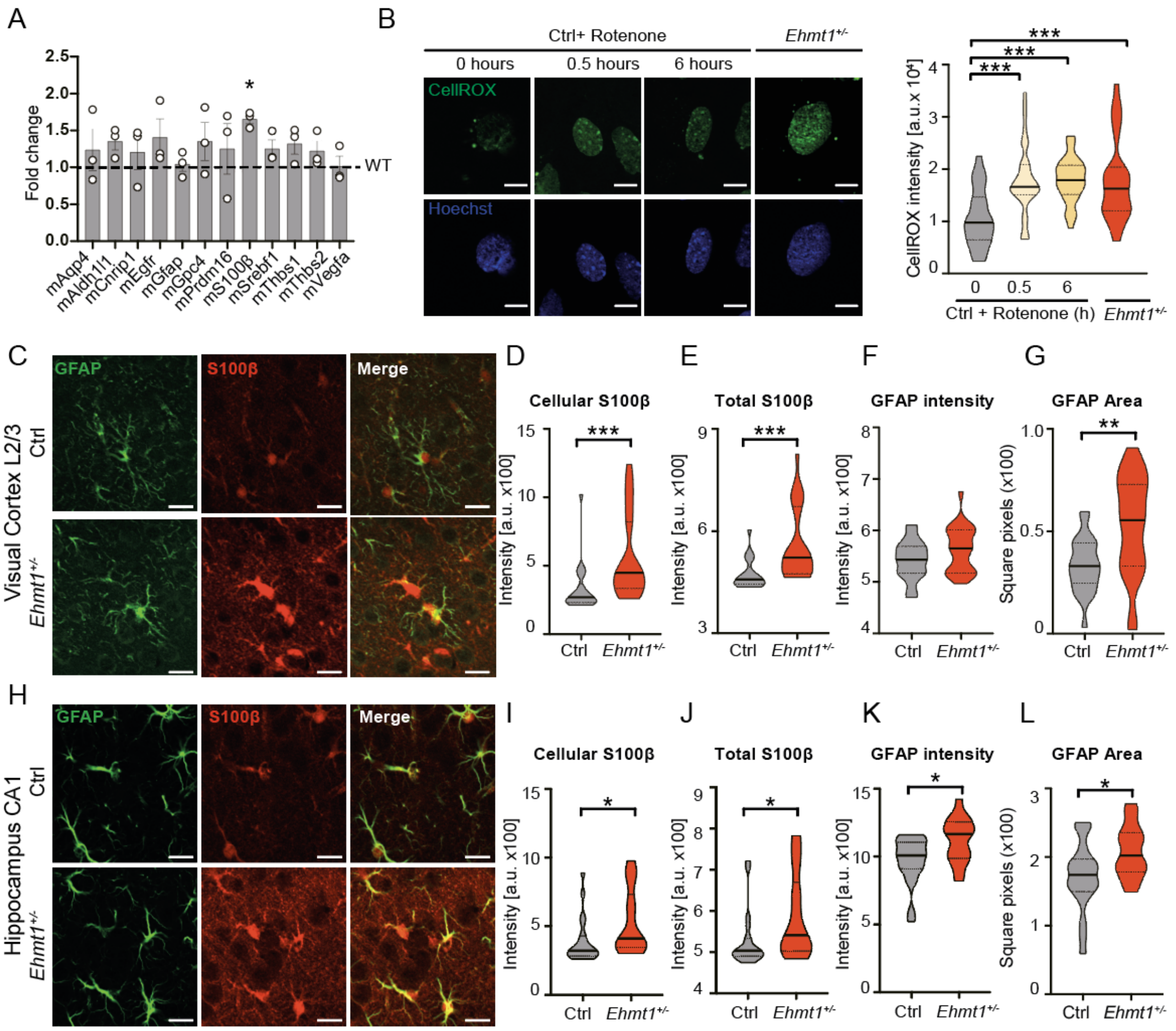

Figure S10. mouse *Ehmt1*$^{+/-}$ astrocytes show a higher level of S100β and produce more reactive oxygen species than control astrocytes in primary cultures and mouse brain slices.

(A) qPCR quantification of *Ehmt1*$^{+/-}$ astrocyte gene expression normalized to control values. Wilcoxon matched-pairs signed rank test with post hoc Bonferroni correction for multiple testing were performed between control and *Ehmt1*$^{+/-}$ conditions (n represents number of litters from which 1 sample of control and *Ehmt1*$^{+/-}$ cultured mice astrocytes were isolated; Expression of *Ehmt1*$^{+/-}$ samples was normalized to the control sample (n = 3 litters). **(B)** Representative images and quantification of CellROX fluorescent intensity in cultured control and *Ehmt1*$^{+/-}$ astrocytes. Two-way ANOVA with post hoc Tukey correction was performed between conditions (N_{cells}/N_{images}: control 0h rotenone n=22/3, control 0.5h 100 nM rotenone n=36/3, control 6h rotenone n=29/3 and *Ehmt1*$^{+/-}$ astrocytes n=29/3 derived from one astrocyte plating and paired rotenone treatment experiment). **(C-G)** Immunofluorescent images **(C)** and quantification of **(D)** intracellular S100β intensity, **(E)** total S100β intensity, **(F)** GFAP intensity and **(G)** GFAP area in layer 2/3 of the Visual Cortex of control and *Ehmt1*$^{+/-}$ mice. **(H-L)** Immunofluorescent images **(H)** and quantification of **(I)** intracellular S100β intensity, **(J)** total S100β intensity, **(K)** GFAP intensity and **(L)** GFAP area in the CA1 of the hippocampus of control and *Ehmt1*$^{+/-}$ mice. Data represent means ± SEM. Unpaired T-test or Mann Whitney U-Test with post hoc Bonferroni correction for multiple testing were performed between control and *Ehmt1*$^{+/-}$ conditions ($N_{images}/N_{litters}$: control CA1 n = 20/3, control VIS layer 2/3 n = 22/3, *Ehmt1*$^{+/-}$ CA1 n = 18/3 and *Ehmt1*$^{+/-}$ VIS layer 2/3 n = 24/3). Data represent means ± SEM. * P<0.05, ** P<0.01, *** P<0.0001. DIV= Days in vitro.

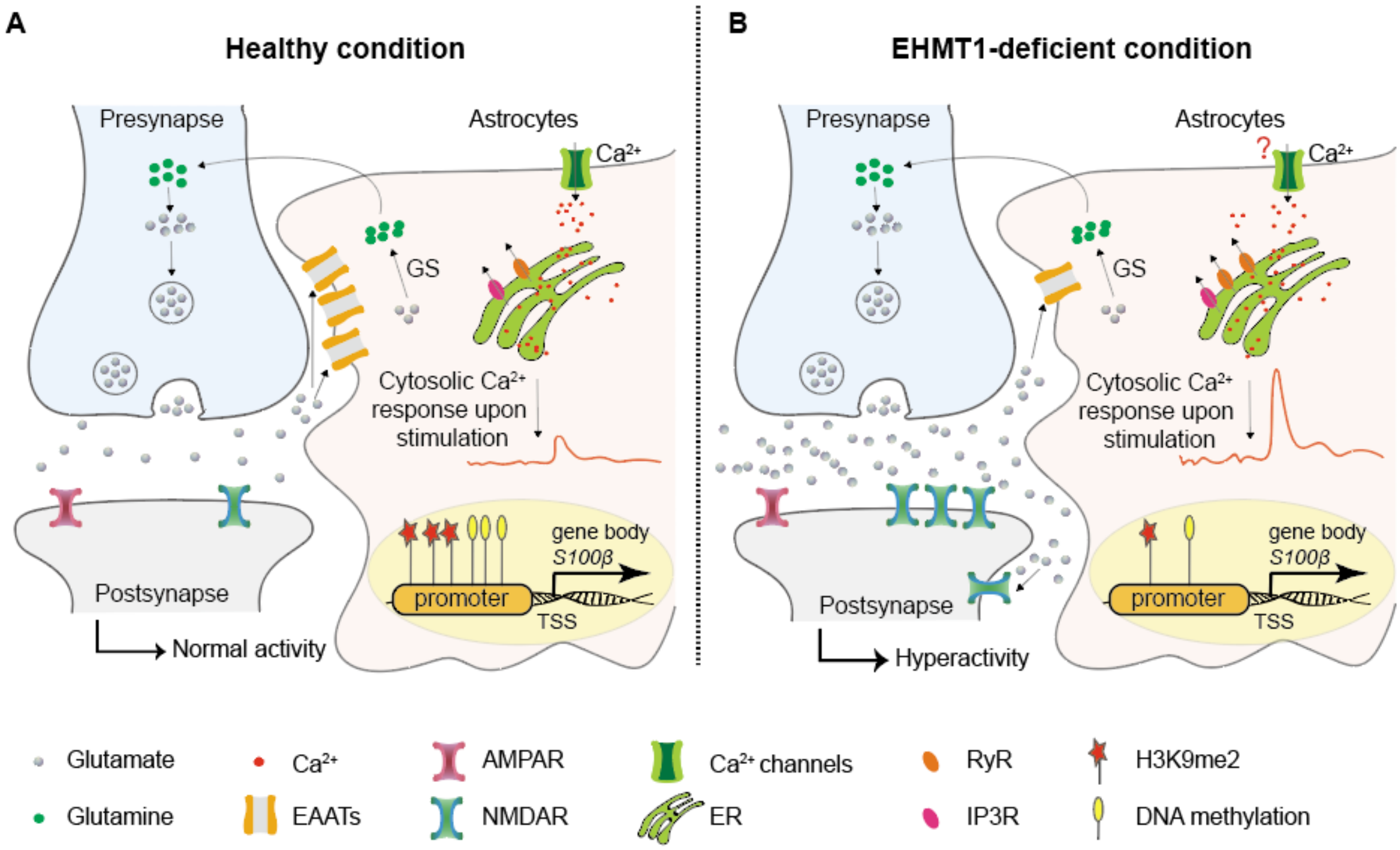

Figure S11. An integrative model of EHMT1-deficient astrocyte-neuron co-cultures. (A) represents the astrocyte-neuronal model in the healthy condition, and **(B)** represents the the EHMT1-deficient condition. *EHMT1*$^{+/-}$ astrocytes show reduced glutamate uptake ability from the synaptic cleft and altered calcium signaling. *EHMT1*$^{+/-}$ astrocytes exhibit reduced expression of genes coding for EAATs (EAAT1; EAAT3; EAAT4 and EAAT5), and altered expression of genes involved in calcium signaling, including increased expression of RyR1 and TRPC4. The transcription of *S100β* in *EHMT1+/-* astrocytes is increased by reduced H3K9me$_2$ mark and DNA methylation at the *S100β* promoter. Increased glutamate level in the synaptic cleft might lead to NMDAR overactivation in neurons, which can cause neuronal hyperactivity and excitotoxicity. Abbreviations: EAAT: excitatory amino acid transporters; AMPAR: α-amino-3-hydroxy-5-methyl-4-isoxazolepropionic acid receptor; GS: glutamine synthase; NMDAR: N-methyl-D-aspartate receptor; ER: endoplasmic reticulum; RyR: ryanodine receptor; IP3R: IP3 receptor; TSS: transcription start site.

Supplementary tables

Supplementary Table 1: Psychiatric diagnoses in published cases with KLEFS1.

Author, year of publication (n=total number of cases)	**Psychiatric disorder(s) conform DSM classification (n= cases with diagnosis)**
Verhoeven, 2011 (n=3)	ASD (n=2), psychotic depression with catatonia (n=1)
Segar, 2015 (n=1)	ASD, OCD, Tourette syndrome,
Schmidt, 2016 (n=8)	ASD (n=8)
Mitra, 2016 (n=1)	ASD, major depressive disorder, psychosis
Vermeulen, 2017 AJMG (n=24)	ASD (n= 22) , MDD (n=10)*, bipolar disorder (n=7)*, psychotic disorder (n=7)*, anxiety disorder (n=11)*, OCD (n=8)*
Taevernier, 2021 (n=1)	ASD, anxiety disorder, MDD, which was changed in bipolar disorder type 1 and later on in psychosis.
*Haseley, 2021** (n=60)*	ASD (n=30), Psychosis (n=7), MDD (n=10), anxiety disorder (n=25), OCD (n=25), hypomania (n=4), catatonia (n=3)
Colijn, 2023 (n=1)	ASD, schizophrenia, bipolar disorder type 1,
Yoshida, 2023	ASD, bipolar disorder type 1, binge eating disorder, addiction (gambling)
Morrison, 2024 (n=103)	Anxiety disorder (n=4), anxiety symptoms without formal diagnosis (n=24), OCD (n=2), OCD symptoms without formal diagnosis (n=25), MDD symptoms without formal diagnosis (n=7)
*Zdolsek Draksler, 2024 ** (n=158)*	Schizophrenia 1,3% (official diagnosis)
Frazier, 2025 (n=65)	ASD 35%

*Lifetime diagnosis. ** parent reported diagnosis, ASD= autism spectrum disorder, MDD= major depressive disorder, OCD= obsessive compulsive disorder.

Supplementary Table 2: Summary of KLEFS1 cases with regression in literature

Reference	Study type (case report, case series, observational cohort, etc.)	Number of included patients (male;female)	Genetic variants	Remarks
Verhoeven, 2011	Case serie	3 (3 f)	2x intragenic LOF mutation*, 1x deletion 9q34.3 **	Patient 1: regression at age 38 years<br>Symptoms: impulsivity and temper tantrums, stiff movements, short periods of non-attention, virtual absence of eye contact, lack of initiative, lack of emotional responsivity<br>Treatment: AF was treated with a B-blocker<br>Patient 2: regression just before 15 years of age with loss of speech and motor function and challenging behavior. Institutionalization was required.<br>Treatment: several psychotropics, including antidepressants as well as antipsychotics (not mentioned which) .<br>Patient 3: regression at age 18 years with gradual deterioration of global functioning, inactivity, loss of energy, decline in speech-language abilities and irritability.<br>Treatment: Aripiprazole 2 mg
Mitra, 2016	Case report	1 (1 m)		Regression at 23 years with severe apathy, catatonia, extreme processing delays, decreased verbal skills, lack of focus on tasks, separation anxiety, aggressive behaviors, depression and severe sleep disorder (disturbed sleep with frequent awakenings and getting up 3-4 times each night). This was accompanied by spontaneous spasms, uncoordinated movements and spluttering. Communication skills deterioriated.<br>His employment was terminated, and he had to move back to his family house.

				Treatment with Olanzapine and additional Desvenlafaxine was effective, and improvement reached almost 100%.<br>The patient was able to return to employment and semi-independent living outside the family house (living home). He is independent in living skills and selfcare again.
Vermeulen et al, 2017 AJMG	Case serie	24 (9m; 15f)	8x intragenic mutation, 16 microdeletions	Lifetime prevalence of regression 50% (12/24). Detailed description and/or treatment is lacking in this publication.
Vermeulen et al, 2017 Neuropharmc	case serie	24 (9m; 15 f),<br>3 detailed cases (3f),<br>same cohort Vermeulen 2017, AJMG	24 (9m; 15 f)<br><br>3 cases<br>1x intragenic mutation; 2x microdeletions	8 patients in cohort (n=24) were above 18 years and all underwent at least 1 episode of developmental regression (8/8; 100% lifetime prevalence above 18 years).<br><br>3 cases:<br>Lost skills comprised speech-language abilities (3), motor skills (3) and social emotional skills (2). The percentage of decline in adaptive functioning ranged from 29-54%.<br><br>Effective treatment included Olanzapine (17,5 mg/d), Aripiprazole (15 mg/d) and Clozapine (50 mg/d).<br><br>Non-effective treatment included Pipamperone (2dd 20mg), benzodiazepines including Lorazepam 4dd 2 mg, Zuclopentixol 50 mg/d and promethazine 50 mg/d)<br>Patient 3 had a partial response on Olanzapine 25 mg and switched to Clozapine, which was effective at a low dosage.
Yoshida et al, 2023	Case report	1 (1m)	1x microdeletion	Behavioral regression is described<br><br>Effective treatment with Risperidone 0,75 mg/d combined with Haloperidol 0,5 mg/d and Clonazepam 0.5-1 mg/d.

Morison, 2024	Case serie	103 (40 m; 63 f)	52x microdeletion, 50x intragenic variant, 1x missing.	11/80 subjects were reported to have shown regression between 1 and 28 years of biological age, including language, social, fine and gross motor skills.

*intragenic loss-of-function mutations: Patient 1: c.3087 þ 1G > T; patient 2: c.3181-80_3233del (p.Tyr1061fs)
** microdeletion minimum size of 0.5 and maximum size of 1.0 Mb in Patient

Supp Table 3: Clinical test battery and procedures

Test	*Test Characteristics*	*Regular visit*	*'Remote' visit*
VABS<br>Vineland-Z NL<br>Vineland III USA	*Vineland Adaptive Behavior Scale* to assess developmental age and possible developmental regression	✓*	✓*
ABAS-3	*Adaptive Behavior Assessment System 3rd edition*	✓	✓
Mini PAS-ADD	*Mini Psychiatric Assessment Schedules for Adults with Developmental Disabilities Screening* instrument for a broad range of psychopathology. Less suitable for negative symptoms	✓	✓
PANSS	*Positive And Negative Syndrome Scale* to measure both positive and negative symptoms	✓	(✓)
ADOS-2	*Autism Diagnostic Observation Schedule* Structured and standardized psychiatric observation and examination for assessing characteristics of autism	✓**	
Beery VMI	Beery Test for Visual-Motor Integration	✓	
LLT	*Location Learning Test* Screen for assessing difficulties with learning and/or difficulties retaining new spatial information	✓	
Cantab<br>Research Suite NL<br>Connect USA	*CAmbridge Neuropsychological Test Automated Battery* Computer tablet based cognitive assessment battery which investigates attention, memory and reaction time	✓	

NL and USA denote which version of the test was used in the Netherlands or the United States of America, respectively. *An additional 'retrospective' analyses when possible. **Only during the first visit. Results not shown in current study.
Primary outcome is adaptive functioning (VABS and/or ABAS). Secondary outcome measures (all others) are used to gain insight into psychiatric disorders at the time of measurement.

During the course of the study, the SARS-CoV-2 pandemic interfered and during lockdowns face to face appointments were not possible. Appointments were therefore changed to 'remote' (i.e. digital) visits. This prompted changes in the regular test battery, as some assessments are not possible when not meeting in real life.

Treatment with olanzapine was initiated when developmental regression was established clinically, combined with either: severe sleep disturbances, disorganized or paranoid behaviors, or negative symptoms (loss of motivation, apathy, slowness). The following regime was used:

- 12-18 years of age or a bodyweight <70 kg: starting dosage of 2,5 mg with a (daily) increase of 2,5 mg until sleep patterns recover. The maximum daily dosage and monitoring were the same as in adult subjects.
- >18 years biological age and >70 kg weight: 10 mg daily with an increase in dosage of +5 mg/day until sleep patterns recover. The maximum daily dosage was 20 mg/daily. Monitoring during treatment was done conform Dutch guidelines for treatment with antipsychotics.
  A lower starting dosage of 5 mg/daily was chosen in case of a history of adverse effects from other psychiatric medication without a known Cytochrome P450 profile.

Supplementary Table 4: cohort characteristics

	Total	Nijmegen	Boston
Gender	54	41	13
Male (%)	19 (35.2)	15 (36.6)	4 (30.8)
Female (%)	35 (64.8)	26 (63.4)	9 (69.2)
Age			
Mean age (years) at inclusion (SD)	25.1 (10.9)	26.2 (12.0)	21.7 (5.8)
Mean developmental age (years) at inclusion (SD)	3.7 (3.4)	3.1 (3.0)	6.7 (4.1)
Genetics			
9q34.3 deletion (%)	16 (29.6)	13 (31.7)	3 (23.1)
Pathogenic *EHMT1* variant (%)	38 (70.4)	28 (68.3)	10 (76.9)

Gender, mean (developmental) age, and genetic variant at inclusion of the cohort.

Supplementary Table 5: number of measurements per participant per age category per assessment, and patient visits

Supplementary Table 6: Scores of VABS subdomains and statistics related to figure 1A.

	**Age category comparison**		
	12-20/ 21-30	*12-20/ 31+*	*21-30/ 31+*
**VABS**			
Communication skills	0,849	**0,002**	**0,001**
Daily living skills	0,608	**0,002**	**<0,001**
Socialization	0,746	**0,001**	**<0,001**
Total	0,726	**<0,001**	**<0,001**

Supplementary Table 7: list of DEG genes in *EHMT1*$^{+/-}$ astrocytes and neurons

Supplementary Table 8: DEGs related to calcium ion binding, calcium signalling and cytokines.

Supplementary Table 9: Primers used in Figure S7A.

Gene	Forward	Reverse
mPrdm16	GAGGTGTCATCCCAGGAGAG	CGAGGTTTTGGTCATCACAG
mS100β	GAAGCCAGAGAGGACTCCAG	CCGGAGTACTGGTGGAAGAC
mGfap	ACGTTAAGCTAGCCCTGGAC	CTGGAGGTTGGAGAAAGTCTG
mAldh1l1	GTGTGTTCACCATCCCAGAC	ACCGAGGGAACTTAAACACG
mAqp4	AATCCAGCTCGATCTTTTGG	ACGTTTGAGCTCCACATCAG
mEgfr	GTGCCACCTGTGTGAAGAAG	GAGCCATGATCTGTCACCAC
mGpc4	TTCAGTGCTCGATTCAGACC	TTGGGAGAGAGGACCAGAAC
mThbs1	TTTACAACGTGGACCAGAGG	CCTCATCGATGTCCTGATTG
mThbs2	GATCGACACAGACAACAATGG	CTCGCTCATTGAAAACATCG
mVegfa	CACAGCAGATGTGAATGCAG	TTTCGTTTTTGACCCTTTCC
mSrebf1	GAGATGTGCGAACTGGACAC	TGGTTGTTGATGAGCTGGAG
mCnrip1	CAACACTGCAGGTCGAGAAC	CGTCAGGCTCTTTACCCTTC
mPpia	AAGGGTTCCTCCTTTCACAG	CCATTATGGCGTGTGAAGTC
mEhmt1	CGTCATAGTGGCCTTTCTTG	CTTGGACGCAGTGAAGTACC

Supplementary table 10: Primers used in Cut & Run targeting human promoter regions.

Gene	Forward	Reverse
Peak 1_primer	TGGGGAAAAGGAGGTTAAATTGTT	TGGGATACATGTTTTATGCCTTTCC
Peak 2_primer	CAGTTGATTGATGATGCTGTTGAGT	ATGAACAGATCCAGCAGATGTGA
Peak 3_primer	TATAGGCGTAAGCCACCCCA	CCACTCTCTTGGACCACAGC
Peak 4_primer	CGAACAAAAGAGGAAAAAGCCC	CTCCACGCTGCTGTTCTCAA
PPIA	TTGGCCTGACCTAATGTTGG	TCCTATGGCTTCCCTTTCAG

Supplementary table 11: Primers used in DNA methylation targeting human promoter regions of *S100B.*

Gene	Forward	Reverse
Primer 1	TGGATGAATTTTGGTGTAGTTTATT	CTTCCTCAACCCATATACAATCCTA
Primer 2	TTATTTTTTTTGTTTAGGGAGGATG	TCCTCAACCCATATACAATCCTAAC
Primer 3	TGGATTGTATTTTAAGGTTTTTTTT	AACAACTAAACTTCTCCTTCCTCAA
Primer 4	TGTGTGGATTGTATTTTAAGGTTTTT	AACAACTAAACTTCTCCTTCCTCAA
Primer 5	TGTGTATATGGTTGTTTTGTTGTTATT	CCACACTACTCAAACTATTCCTCAC
Primer 6	TTGGAAATAGTATAGGGTTGAGAGTT	TATATCAAATCCTTCAACAAAAACC
Primer 7	GTGTGGTTGGTTTTTATTTTTTTAT	TCCAAAACTCAAACTACCACTCTAAA
Primer 8	AGGAATAGTTTGAGTAGTGTGGTTG	CCACTCTAAAAAAATAATCAACACTTACA

Supplemental materials and methods

Methods-Clinical studies

1. Cohort description

Individuals with a genetically confirmed diagnosis of KLEFS1 and a biological age of 12 years and older were recruited from our clinical databases at Department of Human Genetics, Radboudumc Hospital Nijmegen, The Netherlands, as well as the department of Neurology, Boston Children's Hospital, U.S.A. (Supp Table 4).

Informed consent was obtained by legal representatives, since most individuals are incapacitated because of their ID. The regional medical ethical committees of Radboudumc and Boston CH, approved the study under NL65650.091.18 and IRB-P00032696 respectively. Inclusion was done between March 2019 and February 2023. In total 54 individuals participated (41 in Nijmegen and 13 in Boston).

2. Test battery and measures

It is challenging to compile a tailored assessment battery for characterizing cognitive function and psychopathology in individuals with lower intelligence (Vermeulen; et al., 2018). Based on our previous experience we designed a tailored assessment battery applied during the longitudinal course (Supp Table 3). As the primary outcome we measured adaptive functioning for which we used the Adaptive Behavior Assessment System 3rd edition (ABAS-3) and the Vineland Adaptive Behavior Scales (VABS). As these measurements encompass point scales, both absolute and relative comparisons over time, between- and within individuals can be achieved. As secondary outcome measures, we collected data into actual presence of psychiatric disorders by applying the Mini Psychiatric Assessment Schedules for Adults with Developmental Disabilities Screening (mini PAS-ADD) and the Positive And Negative Symptom Scale (PANSS). During the course of the study, the SARS-CoV-2 pandemic interfered with the study design as face-to-face appointments were not possible during lockdowns. Appointments were therefore changed to ‘remote’ (i.e. digital) visits. This prompted alterations in the initial test battery, as some assessments were only possible when meeting life.

Individuals in the Netherlands were visited at home to minimize anxiety and stress from traveling and hospital visits. Individuals in Boston were seen at the outpatient clinic in Boston Children's Hospital. All individuals were visited annually, and in the event of clinical signs of developmental regression or behavioral changes that could indicate the onset of a psychotic episode, the frequency of the visits was doubled to twice per year. Legal representatives or (professional) caregivers served as informants for the (semi-structured) questionnaires/interviews of the test battery and could reach out to the team in case of sudden changes in functioning.

During the start visits, it was explicitly noted whether there was any significant past clinical evidence of experienced regressive or psychotic symptoms. In the case of a positive history of developmental regression or psychotic symptoms, parents were asked to answer the questions on adaptive functioning of the individuals concerned, when their functioning was not yet declined (retrospective analysis). This was supported by the life story and video fragments or photographs to objectify the scores. In several individuals (n=14), data from previous studies or medical/school history, using tests in the current battery (mainly Vineland and Mini PAS-ADD), were available and could be added to the dataset. Further data on age, sex, genotype, and medication use (with special attention to psychopharmacological agents) were collected from (past) medical files.

2a The Vineland Adaptive Behavior Scales (VABS) is a widely used clinical interview, which determines the level of adaptive functioning in ID on the following domains: (1) communication (COM), (2) practical or daily living skills (including motor skills as well as conceptual skills) (DAI) and (3) socialization skills (SOC). In this interview, parents or primary caregivers were interviewed about the individuals. Validity and reliability of this instrument are proven for the specific population with ID (1-4) Different versions of the VABS were used due to varying regional availability (i.e. Vineland-Z in the Netherlands, and the Vineland-3 in the United States).

2b The Adaptive Behavior Assessment System Third Edition (ABAS-3) was used parallel to the VABS. Similar to the VABS, the ABAS-3 assesses the three core domains of adaptive functioning. In addition, it provides a more in-depth assessment of ten skill areas (subdomains). Being a proxy questionnaire, it is filled out by the parents/caregivers of the participant. Three versions are available: for young children aged 0-4 years, for children and adolescents from the age of 5 years up to 17 years, and for adults (aged 18 years up to 80 years) (5,6). Since every patient was 12 years or older, the 'young children aged 0-4 years' version of the ABAS-3 was not used.

2c The mini Psychiatric Assessment Schedules for Adults with Developmental Disabilities (Mini PAS-ADD (Moss et al., 1998; Moss et al., 2008) is a proxy interview for detection of psychiatric symptoms in individuals with developmental disabilities. It consists of 86 items on a 4-points scale (0=absent to 3= severe) and is subdivided into 7 subscales: Major Depressive Disorder, Anxiety, Obsessive-Compulsive disorder, Hypomania/ Mania, Psychosis, Unspecified disorder and Autism. Criteria in this interview are based on the ICD-10 and DSM-IV. This instrument seems to be suitable to assess adolescents with ID, as it has been used before in similar research, and the adjusted criteria for adults with developmental disabilities overlap with adolescents (7). For example, an irritable mood may be an expression of a depressive disorder in adolescents as well as in adults with ID.

2d The Positive And Negative Symptom Scale (PANSS) (8) is an interview to assess the presence or severity of psychotic symptoms in individuals. In particular it is able to capture 'negative' symptoms of psychosis, while the mini PAS-ADD only measures the so-called 'positive' symptoms. It consists of 30 items describing several aspects of psychopathology grouped into three categories (i.e. a positive scale, a negative scale, and a general pathology scale). By subtracting the negative scale from the positive scale, an additional composite scale is established. Each item is scored between 1 (meaning: absent) through 7 (meaning: extreme), based on clearly defined anchoring criteria. Since the theoretical minimum score is 30, prior to the analysis 30 points are deducted from the total score as advocated by Leucht et al (9). While widely used in individuals with schizophrenia in the general population (10), it is also applicable in individuals with (mild) ID to assess psychotic symptoms.

3. Data management

All data was recorded in the CastorEDC database and exported to IBM SPSS™ version 27 for analysis. The acquired data was pseudonymized (with individuals in Nijmegen being coded with an 'N' as a prefix and Boston individuals with a 'B') and entered into Castor Electronic Data Capture (Castor EDC©).

4. Pharmacological intervention

Olanzapine was selected based on literature and clinical case experiences (Supp Table 1). It was prescribed by a local medical doctor according to the following regime and in close collaboration or supervision with the authors (JK, KVK):

· 12-18 years of age or a bodyweight <70 kg: starting dosage of 2,5 mg with a (daily) increase of 2,5 mg until sleep patterns recover. The maximum daily dosage and monitoring were the same as in adult individuals.

· >18 years biological age and >70 kg weight: 10 mg daily with an increase in dosage of +5 mg/day until sleep patterns recover. The maximum daily dosage was 20 mg/daily.

Monitoring during treatment was done according to Dutch guidelines for treatment with antipsychotics. A lower starting dosage of 5 mg/daily was chosen in case of a history of adverse effects from other psychiatric medication without a known cytochrome P450 profile. Local doctors were asked to report on effects, side effects, dosage regime changes and duration of treatment.

Statistical analysis human data

To determine developmental ages from the U.S. individuals, the ABAS-3 results were converted to Vineland-Z scores according to the methods described in Kummeling et al (11).

Statistical analyses were performed on an individual and (sub)group level. Non-parametric testing via Mann-Whitney U and Kruskal-Wallis H was performed for the main analysis on raw scores of the VABS, ABAS-3, Mini PAS-ADD, and PANSS.
Since individuals had varying levels of functioning, ranging from severe to mild ID, the scores were calculated as a percentage based on their initial score at the start of the study.

Neuronal differentiation for serotonergic neurons

WT and *EHMT1*-deficient iPSCs were differentiated into serotonergic neurons by directed conversion. 2 x 10^4 (single) cells/ml were plated at DIV -1 on a 12-well plate and cultured on PLO-mouse laminin (50 µg/ml, Sigma; 20 µg/ml Merck) coated plates throughout the differentiation. Medium is changed complete at the start of each phase (CDM-1 (DIV 0), CDM-2 (DIV 7) and CDM-3 (DIV 14)), and half of the medium is changed once every two days between phases. Cells were split in a 1:3 ratio at the onset of each new phase and supplemented with RevitaCell (1:100, Thermo Fisher Scientific). Single cell hiPSCs were cultured in DIF medium (50% DMEM/F12 (Thermo Fisher Scientific), 50% Neurobasal (Thermo Fisher Scientific), GlutaMAX (1:100, Thermo Fisher Scientific), NEAA (1:100, Thermo Fisher Scientific), N2-supplement (1:100 Thermo Fisher Scientific), B27-supplement (1:50, Thermo Fisher Scientific), primocin (InvivoGen)). From DIV 0 till DIV 6, DIF medium is supplemented with CDM-1 medium (DIF medium + SB431542 (2 µM, Reprocell), DMH1 (2 µM, Tocris) and CHIR99021 (1.4 µM, Reprocell). From DIV 7 till DIV 13, DIF medium is supplemented with CDM-2 medium (CDM-1 + SHH C24II (1 µg/ml, Miltenyi Biotec). From DIV 14 till DIV 20, DIF medium is supplemented with CDM-2 medium (CDM-2 + FGF-4 (10 ng/ml, R&D systems)). On DIV 21, cells are split and cultured onto the MEA (24 well-MEA, Multichannel Systems) at 3 x 10^4 NPCs per well. From DIV 21 onwards, cells are cultured in MAT medium Neurobasal (Thermo Fisher Scientific), GlutaMAX (1:100, Thermo Fisher Scientific), NEAA (1:100, Thermo Fisher Scientific), N2-supplement (1:100 Thermo Fisher Scientific), B27-supplement (1:50, Thermo Fisher Scientific), primocin (InvivoGen) supplemented with ascorbic acid (200 µM, Merck), DAPT (2.5 µM, Merck) , GDNF (10 ng/ml, PeproTech), BDNF (10 ng/ml, PromoKine), IGF-I (10 ng/ml, PeproTech, TGF-β3 (1 ng/ml, Merck) and mouse laminin (10 ng/ml, Merck). On DIV 23, mouse primary astrocytes are added to the culture. AraC (2 µM, Sigma) is added to the medium between DIV 28 and 35 to remove remaining NPCs from the culture. Neuronal cultures were kept through the whole differentiation process at 37°C/ 5% CO_2.

Neuronal differentiation for dopaminergic neurons

WT and EHMT1-deficient hiPSCs were differentiated into dopaminergic neurons as described previously (12). 4-8 x 10^4 (single) cells/ml were plated at DIV -2 on a 12-well plate and cultured

on vitronectin (1:100, Thermo Fisher Scientific) coated plates throughout the differentiation. The medium was supplemented with RevitaCell (1:100, Thermo Fisher Scientific) after each split into single cells. During the differentiation, split the cells into single cells in a ratio of 1:2/1:3 when very confluent and add thiazovivin (2 µM, Sigma) to the medium. On DIV -1, the iPSC culture medium (E8 Flex, Thermo Fisher Scientific) is refreshed to remove RevitaCell. From DIV 0 till DIV 5, the cells are cultured in KO DMEM complete medium (KO DMEM (Thermo Fisher Scientific), NEAA (1:100 Merck), GlutaMAX (1:100, Thermo Fisher Scientific, primocin (InvivoGen), KO serum replacement (15%, Thermo Fisher Scientific)). On DIV 0, the medium is changed to DIV0 medium (KO DMEM complete medium + mecaptoethanol (0.1 mM, Merck), LDN-193189 (100 nM, Reprocell), SB431542 (10 µM, Reprocell). On DIV 1, the medium is changed to DIV1 medium (DIV0 medium + Purmorphamine (2 µM, Reprocell), FGF-8a (0.1 µg/ml, R&D systems). The medium for DIV2-4 is changed every day with DIV2 medium (DIV 1 medium + CHIR99021 (3 µM, Reprocell). On DIV 5, prepare N2 medium (DMEM/F12 (Thermo Fisher Scientific), primocin, N2 (1:100, Thermo Fisher Scientific)). From DIV 5 till DIV 7, the medium is changed gradually (steps of 25%) to N2 medium (DIV 5: 75%-25%, DIV 6: 50%-50%, DIV 7: 75%-25%) and is supplemented with mecaptoethanol (0.1 mM), LDN-193189(100 nM), Purmorphamine (2 µM), FGF-8a (0.1 µg/ml), CHIR99021 (3 µM) and SHH (0.1 µg/ml). From DIV 8 till DIV 10, the medium is changed every day with 100% N2 medium supplemented with LDN-193189(100 nM), Purmorphamine (2 µM), FGF-8a (0.1 µg/ml), CHIR99021 (3 µM) and SHH (0.1 µg/ml). On DIV 11, change the medium for DIV 11 medium (NB complete, BDNF (20 ng/ml, PromoKine), GDNF (20 ng/ml, PeproTech), cAMP (0.5mM, Enzo Life Sciences), TGF-beta 3 (2 ng/ml, Merck), Ascorbic acid (200 µM, Merck), DAPT (10 nM, Merck), CHIR99021 (3 µM). On DIV 12, prepare NB complete medium (Neurobasal (Thermo Fisher Scientific), Glutamax (1:100), primocin, B27 (1:50, Thermo Fisher Scientific). From DIV 12 till DIV 19, only half of the medium is changed once every two days for growth medium (NB complete, BDNF (20 ng/ml), GDNF (20 ng/ml), cAMP (0.5mM), TGF-beta 3 (2 ng/ml), Ascorbic acid (200 µM), DAPT (10 nM). On DIV 19, split the cells and plate 1.5-3 x 104 cells per 24-well plate coated with PLO-mouse laminin (50 µg/ml, Merck; 20 µg/ml, Merck) and culture them in growth medium supplemented with thiazovivin (2 µM). On DIV 21, change medium to dopaminergic medium (NB complete, BDNF (20 ng/ml), GDNF (20 ng/ml), cAMP (0.5 mM), TGF-beta 3 (2 ng/ml), Ascorbic acid (200 µM), DAPT (10 µM, note that DAPT concentration increased from growth medium)). On DIV 23 mouse primary astrocytes are added to the culture. From DIV 24 onwards, change half of the medium once every two days for dopaminergic medium. Neuronal cultures were kept through the whole differentiation process at 37°C/ 5% CO_2.

Cut & Run and RT-PCR

Cut & Run was performed using the CUT & RUN assay kit (Cell signalling technology, 86652) according to the datasheet. In brief, cells were resuspended in wash buffer supplemented with spermidine and protein inhibit cocktail (PIC). Activated Concanavalin A Magnetic beads were added to the cell suspension in a low DNA binding tube. 5 μg H3K9me2 antibody (Abcam, ab1220) was added into each tube with antibody binding buffer and rotated at 4℃ overnight. Primer sequences can be seen from Supplementary table 9. pAG-MNase enzyme was added to each tube and rotated at 4℃ for 1 hour. 150 μl of digitonin buffer (+ spermidine + PIC + digitonin) was added to each tube and mixed. pAG-MNase was activated by adding 3 μl cold calcium chloride. Samples were centrifuged at 4°C for 2 min at 16,000x g and supernatans transferred to new 2 ml microcentrifuge tubes. DNA was purified by QIAamp DNA Mni Kit (QIAGEN, 51304) according to the instructions on data sheet. RT-PCR was subsequently performed in the end. Percent Input = 100% x 2(C[T] 100%Input Sample - C[T] IP Sample).

Glutamate uptake

Medium were collected from the iPSC-derived astrocyte cultures at 3 hours after the addition of glutamate (100 μM). After this, medium was diluted in a proper ratio (~1:50). Glutamate concentrations in the collected samples were measured using the Amplex™ Red Glutamic Acid Assay Kit (Thermo Fisher, A12221) according to the manufacturer instructions. Fluorescent signal was measured at 540 nm excitation and 590 nm emission using a microplate reader (Tecan's Spark® 20M multimode reader). Total protein concentration of the astrocyte culture was measured using the Pierce™ BCA Protein Assay Kit (Thermo Fisher, 23225) according to the manufacturer instructions. Glutamate uptake by each astrocyte culture was normalized for the amount of protein in the cell culture to calculate the glutamate uptake (μM) per μg protein.

Calcium imaging

To prepare the cells for calcium imaging, cultured astrocytes were incubated in HBSS with 4 μg/ml Fluo-8-AM (Abcam, ab142773) for 30 min at 37°C. Next, three continued washing steps with HBBS removed the excess dye in the medium. Finally, astrocytes were left in culture medium to recover for 15 min. For imaging the cells, we used a SliceScope Pro 2000 Scientifica microscope with a 20X magnification combined with the sCMOS camera (Prime BSI Express, Teledyne Photometrics). During the recordings, the cells were continuously perfused with oxygenated ACSF (95% O_2/ 5% CO_2) containing 124 mM NaCl, 3 mM KCl, 1,25 mM NaH_2PO_4, 2 mM $CaCl_2$, 1 mM $MgCl_2$, 26 mM $NaHCO_3$ and 10 mM Glucose at 37°C. For the imaging, cells were excited at 470nm by LED (KSL470, Rapp OptoElectronic) and frame

scans were acquired at 4 Hz for a period of 20 minutes for the evoked response and 10 minutes for the spontaneous response. To analyze the evoked response of the astrocytes, a first recording was performed in the previously described ACSF so as to assess the baseline fluorescence. After 2 minutes, the original ACSF was replaced with ACSF containing 50 μM glutamate perfused slowly into the medium and the region was recorded for 10 minutes.

After the recordings, the videos were analyzed using a home-made script in Matlab (The Math Works, Inc. MATLAB. Version 2020b). The ROIs had been selected manually following the shape of the corresponding astrocyte to analyze from a static image, preventing any possible bias made towards active cells. The calcium response signal was obtained as a change in the fluorescence of the ROI according to the formula $\Delta F/F = (F-F0)/F0$; where F0 represents the baseline fluorescence and F the fluorescence of each ROI over time. In addition, the decay of the baseline intensity due to bleaching was corrected by exponential fitting.

ELISA kit for S100B

Medium was collected 48 hours after changing medium. After this, medium was diluted in a proper ratio. S100B was tested using an ELISA kit (R&D, DY1820-05). Total protein concentration of the astrocyte culture was measured using the Pierce™ BCA Protein Assay Kit (Thermo Fisher, 23225) according to the manufacturer instructions. S100B concentration in each astrocyte culture was normalized for the amount of protein in the cell culture.

S100B RNA interference

HEK293T cells (ATCC, CRL-3216) were cultured in high-glucose DMEM (Sigma, D0819) supplemented with 10% Sodium-Pyruvate (Sigma, S8636), 1% Pen/strep and 10% FCS in 10 cm petri dishes. Cells were split using 0.05% Trypsin-EDTA (GIBCO, 25300054) in 1:10 dilution for the generation of lentiviral particles, using four plates per construct. For RNA interference (RNAi) knockdown experiments, a DNA fragment encoding a short hairpin RNAs (shRNA) directed against S100β was cloned into a lentiviral vector by VectorBuilder (pLV[shRNA]-m-cherry: T2A: Puro-U6>hS100β[shRNA#3], vector ID: VB900129-7961wcj). The S100β target sequence was: AGCAGGAGGTTGTGGACAAAG. A scrambled shRNA was used as non-targeting control, cloned into a lentiviral vector by VectorBuilder (pLV[shRNA]-m-cherry: T2A: Puro-U6>Scramble_shRNA#1) with the following hairpin sequence: CCTAAGGTTAAGTCGCCCTCG. Lentiviruses were generated by co-transfecting each lentiviral vector, the psPAX2 packaging vector (Addgene, #12260) and the VSVG envelope glycoprotein vector pMD2-G (Addgene, #12259) into HEK293T cells, using calcium phosphate precipitation. The supernatant of culture medium was collected 48 hours after transfection and filtered through a 0.45 μm syringe filter. Lenti-X Concentrator (Takara, 631231) was added in a 1:4 ratio to the filtered medium, which was incubated overnight at 4°C. After centrifugation

the supernatant was removed and the cell pellet was dissolved in PBS. Viral particles were stored at -80°C.

DNA methylation and primer design

For primer design, we chose eight potential DNA methylation sites in the promoter region of S100B based on ENCODE database. We used MethPrimer (online software) to design eight different primer pairs primers (Supplemental Table 11). Sodium bisulfite conversion was performed using a commercial DNA methylation kit (ZYMO, D5030) following the data sheet. In brief, genomic DNA was extracted using Protein kinase K, after which DNA was purified using QIAamp DNA Mini kit (QIAGEN, 51304). 500 ng to 1 µg genomic DNA was used as starting material for bisulfite conversion according to the manufacturer's protocol. Subsequently, PCR was performed to amplify the fragments including the potential DNA methylation sites. Sodium bisulfite converts unmethylated cytosine to uracil which is replicated as thymine in the subsequent PCR step. Cleaned-up PCR product was sent for Sanger Sequence. Methylation percentages at specific CpG sites were determined as the cytosine peak height (Forward primer) or guanine peak height (Reverse primer), divided by the peak height sum of cytosine and thymine (Forward primer) or guanine and adenine (Reverse primer) (Supplemental Table 11).

Supplemental references

1. de Bildt A, Kraijer D, Sytema S, Minderaa R (2005) The psychometric properties of the Vineland Adaptive Behavior Scales in children and adolescents with mental retardation. J Autism Dev Disord 35:53–62.
2. Sparrow, S., Balla, D., & Cicchetti, D. V. (1984). Vineland Adaptive Behavior Scales: Survey edition. In. Circle Pines, MN: American Guidence Service.
3. Sparrow, S. S., Balla, D. A., Cicchetti, D. V., & de Bilt, A. A. K. D. W. (2009). Vineland-Z. In *Sociale redzaamheidsschaal voor kinderen en jeugdigen met een verstandelijke beperking*. Leiden: PITS B.V.
4. Sparrow, S. S., C; Cicchetti, D; Doll, E. . (2016). Vineland 3: Vineland adaptive behavior scales. In. Minneapolis, MN: Pearson Assessments.
5. Harrison, P. L., & Oakland, T. (2015a). ABAS-3 Schaal voor Adaptief Gedrag. Versie voor Volwassenen. In. Amsterdam, Netherlands: Hogrefe Uitgevers BV.
6. Harrison, P. L., & Oakland, T. (2015b). ABAS-3: Adaptive behavior assessment system. In: Western Psychological Services.
7. Vermeulen, K., de Boer, A., Janzing, J. G. E., Koolen, D. A., Ockeloen, C. W., Willemsen, M. H., Verhoef, F. M., van Deurzen, P. A. M., van Dongen, L., van Bokhoven, H., Egger, J. I. M., Staal, W. G., & Kleefstra, T. (2017). Adaptive and maladaptive functioning in Kleefstra syndrome compared to other rare genetic disorders with intellectual disabilities. *Am J Med Genet A*, *173*(7), 1821-1830.
8. Kay SR, Fiszbein A, Opler LA.The positive and negative syndrome scale (PANSS) for schizophrenia. 13:261–276.
9. Stefan Leucht, Werner Kissling, John M. Davis, (2010) The PANSS Should Be Rescaled, *Schizophrenia Bulletin*,. 36:461–462.
10. Obermeier M, Schennach-Wolff R, Meyer S, Möller HJ, Riedel M, Krause D, Seemüller F. Is the PANSS used correctly? a systematic review. BMC Psychiatry. 2011 11:113.
11. Kummeling, J., Vermeulen-Kalk, K., Souverein, V., van Dongen, L. C. M., Oomens, W., Janzing, J. G. E., Pop-Purceleanu, M., Kleefstra, T., & Egger, J. I. M. (2023). Measuring Adaptive Behavior in Patients with Mendelian Neurodevelopmental Disorders. Comparison of ABAS-3 and Dutch Vineland Scales. *Clin Neuropsychiatry*, *20*(5), 453-461
12. Van Rhijn J-R, Shi Y, Bormann M, Mossink B, Frega M, Recaioglu H, Hakobjan M, Klein Gunnewiek T, Schoenmaker C, Palmer E, Faivre L, Kittel-Schneider S, Schubert D, Brunner H, Franke B, Nadif Kasri N.Brunner syndrome associated MAOA mutations result in NMDAR hyperfunction and increased network activity in human dopaminergic neurons. *Neurobiol. Dis.* 2022;163:105587.
13. Kim N Wijnant, Imke ME Schuurmans, Annika Mordelt, Ka Man Wu, Michael Kwint, Lisenka ELM Vissers, Nael Nadif Kasri (2026) Mapping NMD-coupled alternative splicing in iPSC-derived brain cells: a resource for therapeutic discovery bioRxiv 2026.01.19.700316; doi: https://doi.org/10.64898/2026.01.19.700316
14. Frega M et al. Neuronal network dysfunction in a model for Kleefstra syndrome mediated by enhanced NMDAR signaling. Nat Comms 2019;10(1):4928.
