# Supplementary material for: Astrocytes contribute to olanzapine-mediated reversal of kleefstra syndrome-associated neurodevelopmental regression
Source: J Clin Invest. 2026 Jun 30;136(16):e195803. doi: 10.1172/JCI195803 (PMC13476134; doi:10.1172/JCI195803)

Blots belinging to figure 2

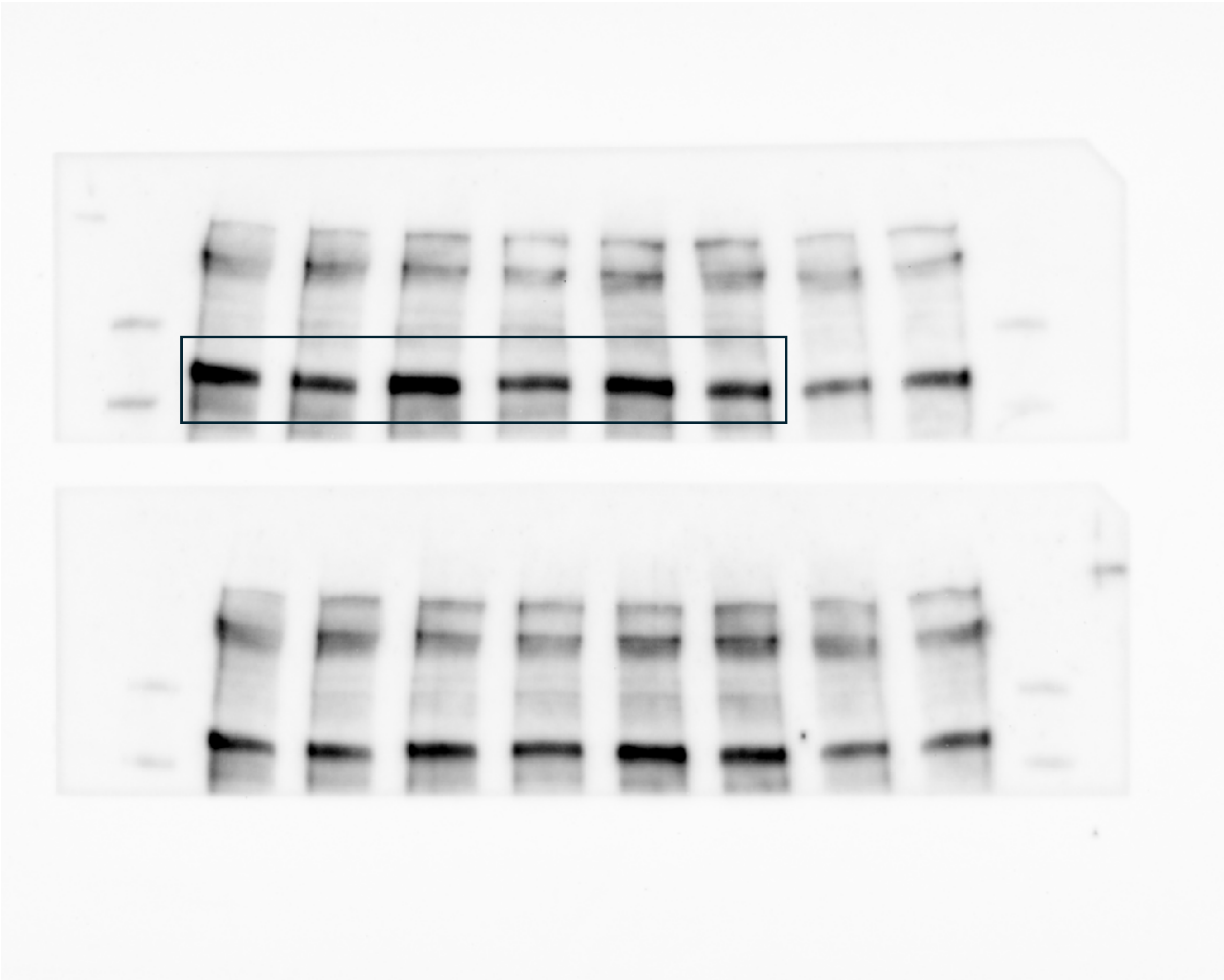

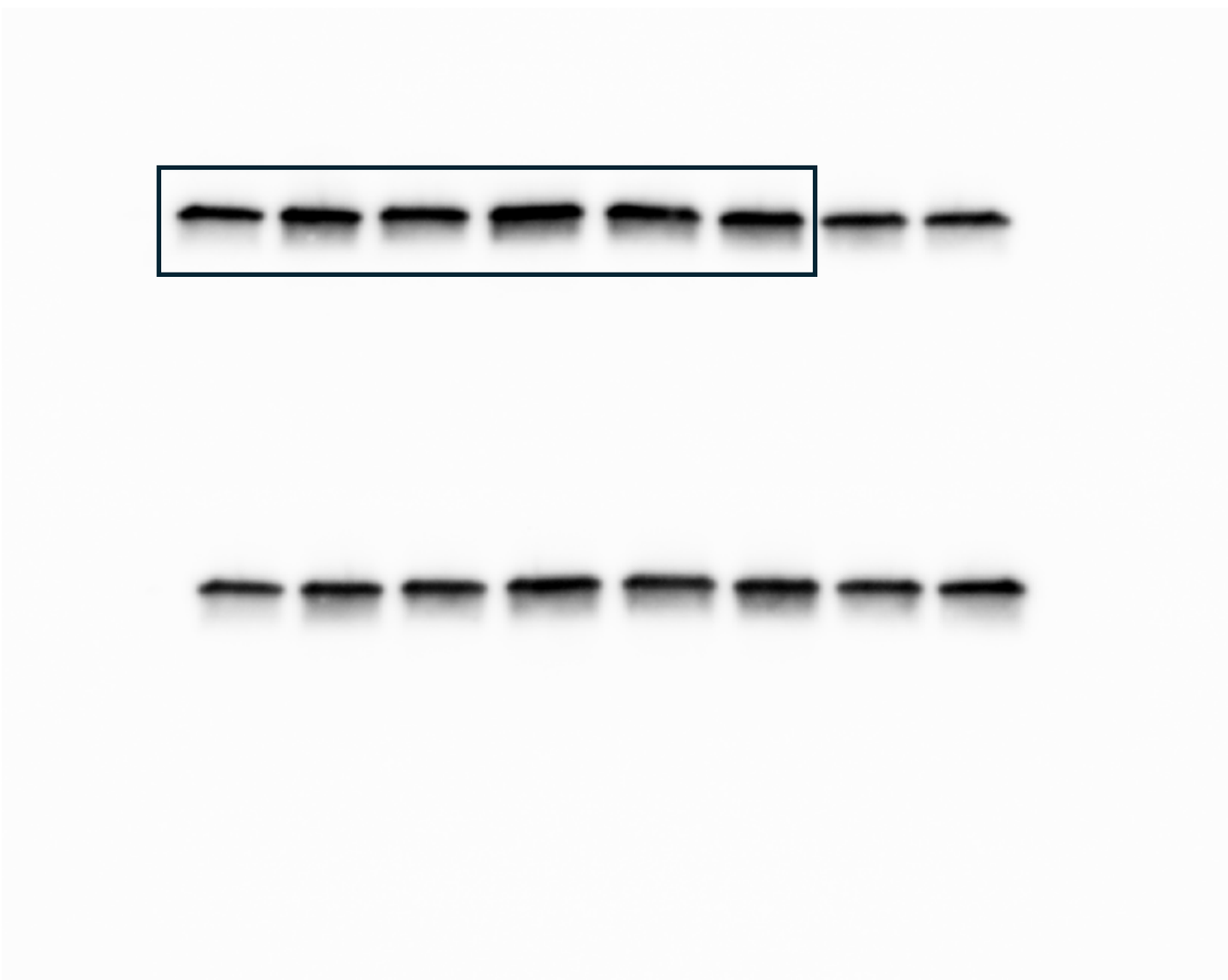

Blots belonging to figure 4C - olanzapine

JAK: blot was cut before blotting to save Ab costs and blot against several proteins

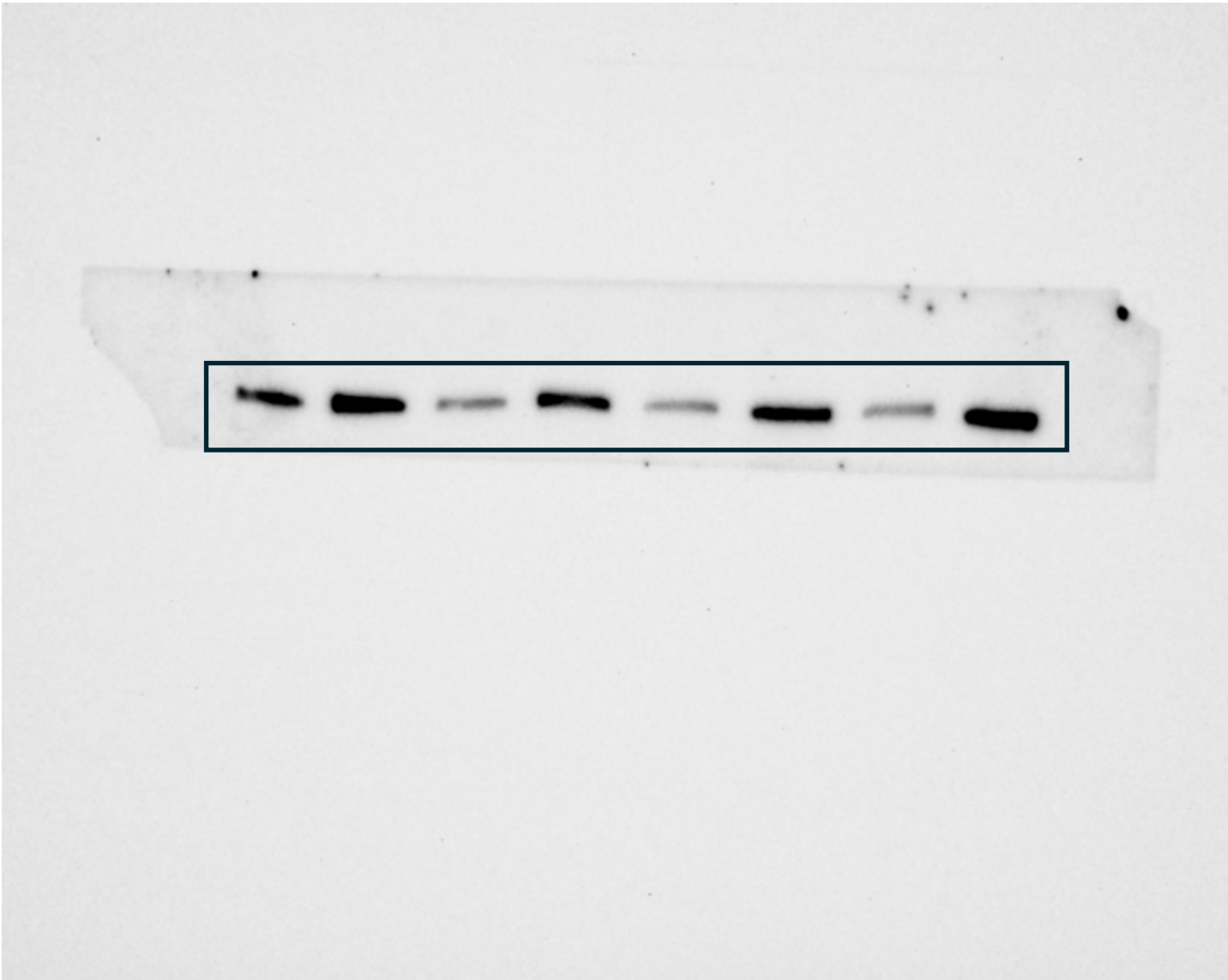

STAT3: blot was cut before blotting to save Ab costs and blot against several proteins

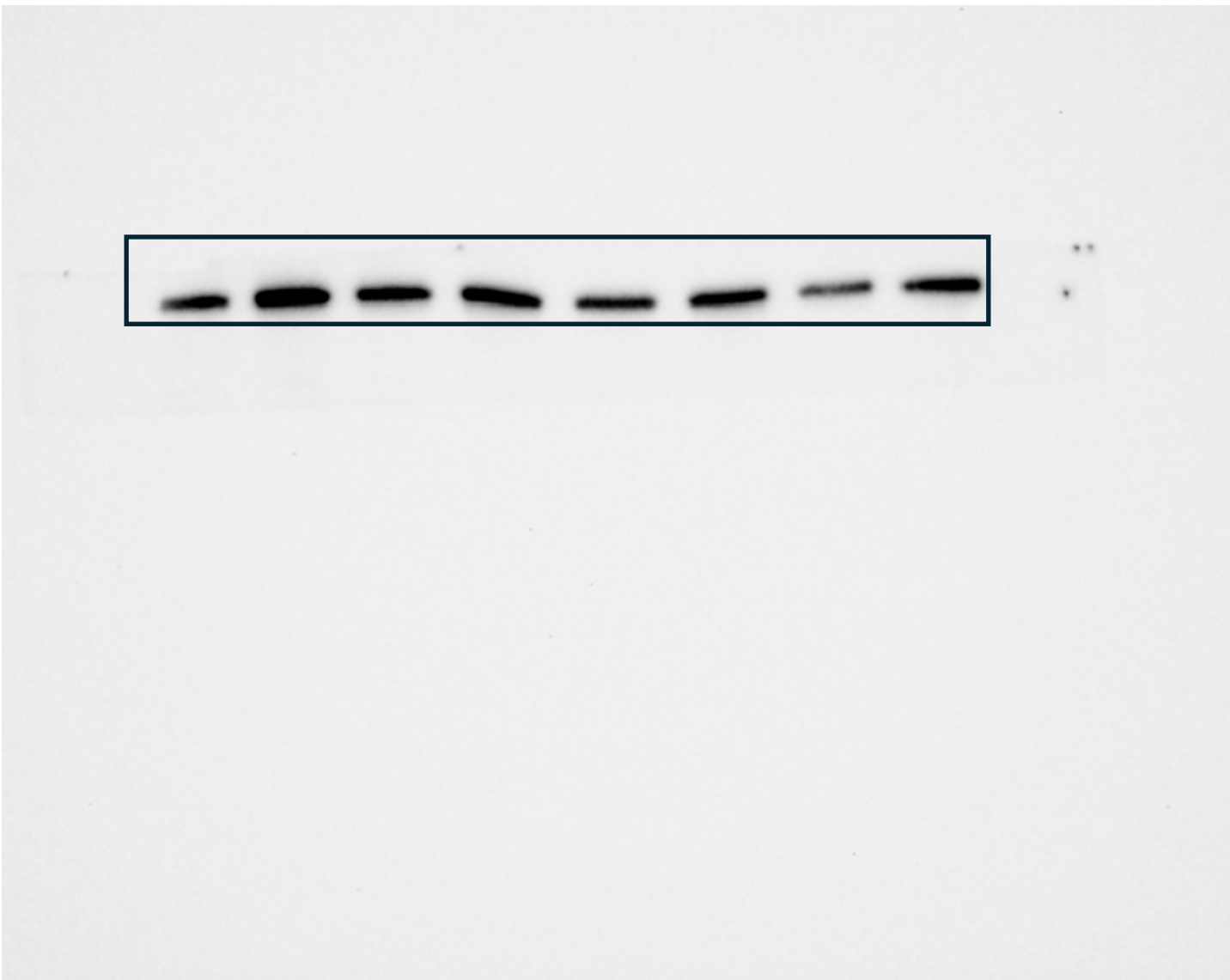

GAPDH: blot was cut before blotting to save Ab costs and blot against several proteins

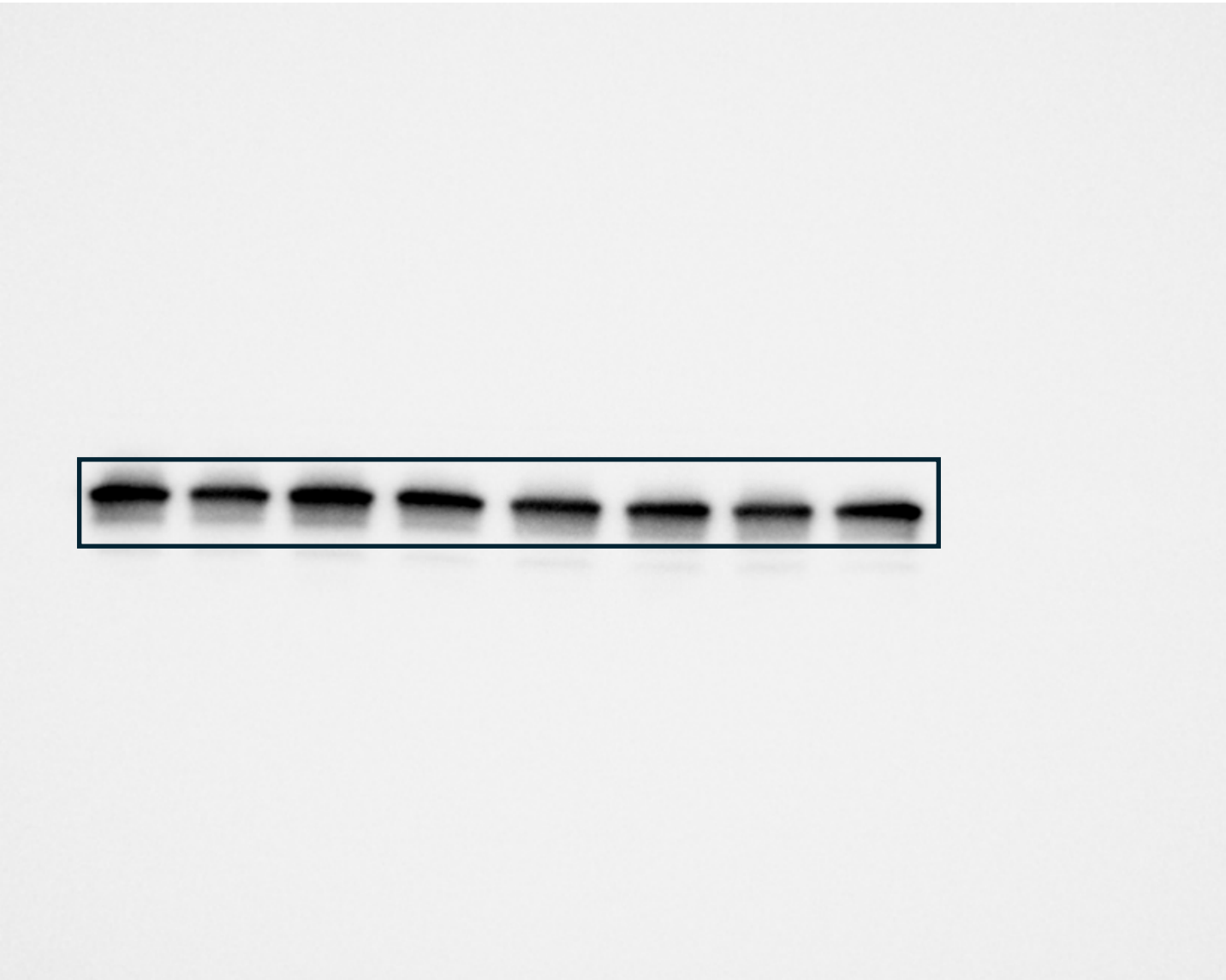

Fig 4C + Olanzapine STAT3

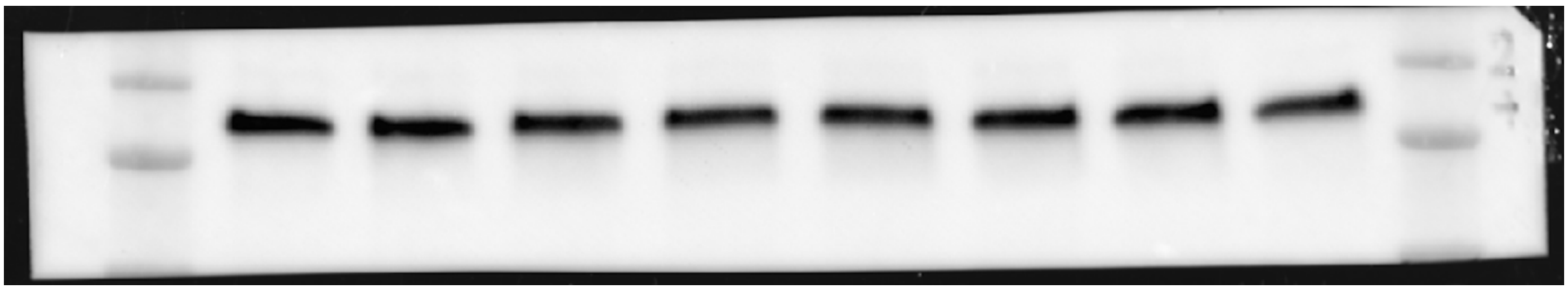

Fig 4C + Olanzapine JAK

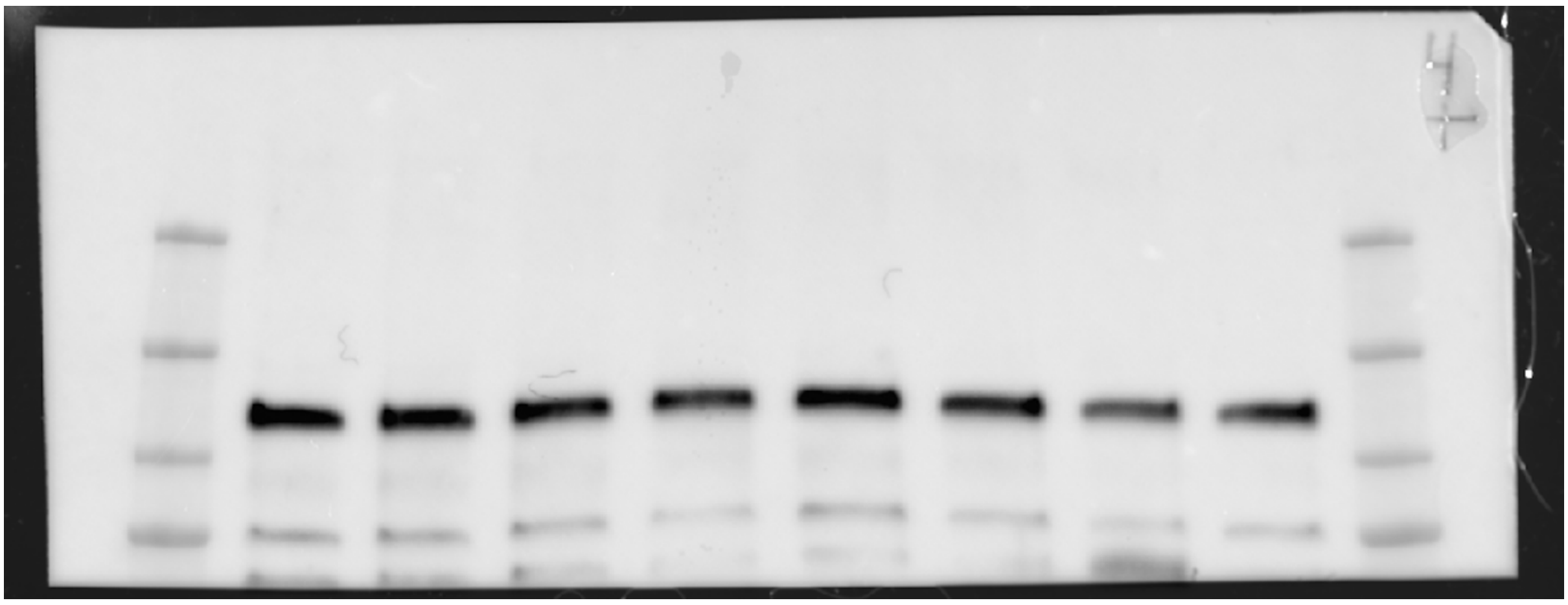

GAPDH

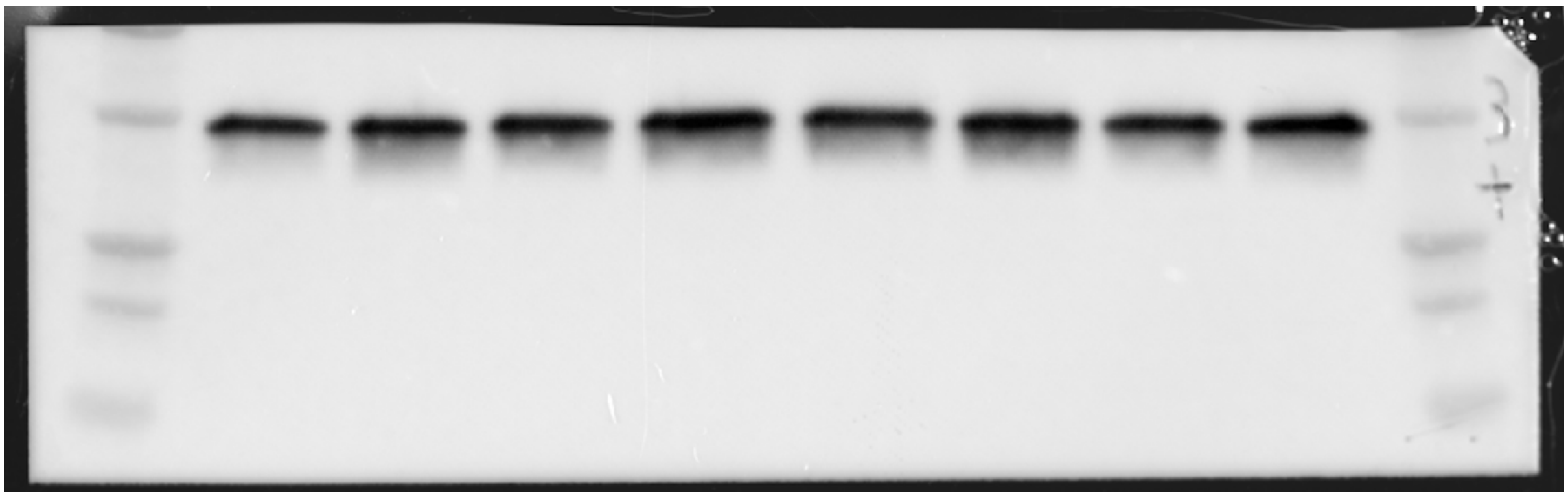

Supplement: Supplementary file 2 [file jci-136-195803-s071.pdf]
